# ITIH5 mediates epigenetic reprogramming of breast cancer cells

**DOI:** 10.1186/s12943-017-0610-2

**Published:** 2017-02-23

**Authors:** Michael Rose, Vera Kloten, Erik Noetzel, Lukas Gola, Josef Ehling, Timon Heide, Steffen K. Meurer, Aljona Gaiko-Shcherbak, Antonio S. Sechi, Sebastian Huth, Ralf Weiskirchen, Oliver Klaas, Wiebke Antonopoulos, Qiong Lin, Wolfgang Wagner, Jürgen Veeck, Felix Gremse, Julia Steitz, Ruth Knüchel, Edgar Dahl

**Affiliations:** 1 Institute of Pathology, Medical Faculty of the RWTH Aachen University, Aachen, Germany; 2 Institute of Complex Systems, ICS-7: Biomechanics, Forschungszentrum Jülich GmbH, Jülich, Germany; 3 Department of Experimental Molecular Imaging (ExMI), Helmholtz Institute for Biomedical Engineering, Medical Faculty of the RWTH Aachen University, Aachen, Germany; 4 Experimental Gene Therapy and Clinical Chemistry, Institute of Molecular Pathobiochemistry, Medical Faculty of the RWTH Aachen University, Aachen, Germany; 5 Institute for Biomedical Engineering—Cell Biology, Medical Faculty of the RWTH Aachen University, Aachen, Germany; 6 Helmholtz-Institute for Biomedical Engineering—Stem Cell Biology and Cellular Engineering, Medical Faculty of the RWTH Aachen University, Aachen, Germany; 7 Division of Medical Oncology, Department of Internal Medicine, Department of Pathology, GROW-School for Oncology and Developmental Biology, Maastricht University Medical Centre, Maastricht, The Netherlands; 8 Institute for Laboratory Animal Science, Medical Faculty of the RWTH Aachen University, Aachen, Germany

**Keywords:** ITIH5, Breast cancer, Extracellular matrix, Epigenetic reprogramming, Cancer stem cells, DAPK1

## Abstract

**Background:**

Extracellular matrix (ECM) is known to maintain epithelial integrity. In carcinogenesis ECM degradation triggers metastasis by controlling migration and differentiation including cancer stem cell (CSC) characteristics. The ECM-modulator inter- α-trypsin inhibitor heavy chain family member five (ITIH5) was recently identified as tumor suppressor potentially involved in impairing breast cancer progression but molecular mechanisms underlying its function are still elusive.

**Methods:**

*ITIH5* expression was analyzed using the public TCGA portal. ITIH5-overexpressing single-cell clones were established based on T47D and MDA-MB-231 cell lines. Colony formation, growth, apoptosis, migration, matrix adhesion, traction force analyses and polarization of tumor cells were studied in vitro. Tumor-initiating characteristics were analyzed by generating a metastasis mouse model. To identify ITIH5-affected pathways we utilized genome wide gene expression and DNA methylation profiles. RNA-interference targeting the ITIH5-downstream regulated gene *DAPK1* was used to confirm functional involvement.

**Results:**

*ITIH5* loss was pronounced in breast cancer subtypes with unfavorable prognosis like basal-type tumors. Functionally, cell and colony formation was impaired after ITIH5 re-expression in both cell lines. In a metastasis mouse model, ITIH5 expressing MDA-MB-231 cells almost completely failed to initiate lung metastases. In these metastatic cells ITIH5 modulated cell-matrix adhesion dynamics and altered biomechanical cues. The profile of integrin receptors was shifted towards β1-integrin accompanied by decreased Rac1 and increased RhoA activity in ITIH5-expressing clones while cell polarization and single-cell migration was impaired. Instead ITIH5 expression triggered the formation of epithelial-like cell clusters that underwent an epigenetic reprogramming. 214 promoter regions potentially marked with either H3K4 and /or H3K27 methylation showed a hyper- or hypomethylated DNA configuration due to ITIH5 expression finally leading to re-expression of the tumor suppressor DAPK1. In turn, RNAi-mediated knockdown of DAPK1 in ITIH5-expressing MDA-MB-231 single-cell clones clearly restored cell motility.

**Conclusions:**

Our results provide evidence that ITIH5 triggers a reprogramming of breast cancer cells with known stem CSC properties towards an epithelial-like phenotype through global epigenetic changes effecting known tumor suppressor genes like DAPK1. Therewith, ITIH5 may represent an ECM modulator in epithelial breast tissue mediating suppression of tumor initiating cancer cell characteristics which are thought being responsible for the metastasis of breast cancer.

**Electronic supplementary material:**

The online version of this article (doi:10.1186/s12943-017-0610-2) contains supplementary material, which is available to authorized users.

## Background

Turnover of the extracellular matrix (ECM) is a critical step in various aspects of tumor cell biology, e.g. in orchestrating breast cancer cell differentiation driving malignancy and metastasis [1, 2]. Inter-α-trypsin inhibitory (ITI) proteins comprise a family of secreted serine protease inhibitors found in both the ECM and in the blood circulation [3]. ITIs are composed of a light chain, also called Bikunin, and different homologous heavy chains (i.e. ITIHs). ITIHs are covalently linked to Bikunin and thereby form a structural and functionally unique protein with a plasma protease inhibitory activity [4]. Beyond this the biological function of ITI heavy chains remains largely unknown. Trimming of precursor ITIH proteins at a conserved cleavage site unmasks a C-terminal amino acid [4], which is involved in hyaluronic acid (HA) binding [5]. Owing to that ITI heavy chains were originally referred to as serum-derived HA associated proteins (SHAPs) [6], implicating a wide spectrum of biological activities. HA that is the major proteoglycan of the ECM interacts with a large number of HA-binding proteins (HABPs) [4] like HA-receptors CD44 and RHAMM [7, 8]. Unlike all other described HABPs, ITI heavy chains are covalently linked with HA [3], whose complexation generate stable “cable-like structures” supporting ECM integrity. In 1994 Chen and colleagues showed that ITI heavy chains are involved in organizing and controlling of the cumulus-oocyte expansion [9]. In carcinogenesis of various tumor entities, accumulating studies propose a tumor suppressive role of ITI heavy chains mediated by their ECM-stabilizing activity [10–12]. ITIH1 and ITIH3, for instance, have been demonstrated to cause clear retardation of lung metastasis in vivo [12] thereby suggesting an important role of ITI heavy chains in repressing malignant diseases independently of Bikunin.

In 2004 we identified ITIH5 as the fifth heavy chain member of the ITI family [13]. ITIH5 contains all structural features found in ITIH1-3, including distinct functional domains (VIT and vWA) and the conserved cleavage site. Nevertheless, its expression pattern differs from that of other heavy chains, i.e. ITIH5 is abundantly expressed in the placenta and moderately expressed in various organs such as the mammary gland [13] indicating a local, tissue-specific function. ITIH5 dysfunction has been shown to contribute to inflammatory skin diseases [14] and obesity, thus potentially acting as regulator of human metabolism [15]. In tumor development, downregulation of ITIH5 caused by aberrant DNA hypermethylation has been reported in breast cancer [16, 17], bladder cancer [18], colon cancer [19], gastric cancer [20] and lung cancer [21]. Based on an integrated genomic and transcriptomic approach Wu and colleagues recently demonstrated rare somatic *ITIH5* gene mutations in lung cancer whose frequency increased up to 6% in corresponding metastases [22]. Loss of ITIH5 expression in breast and bladder cancer has been associated with clinical parameters of malignant progression and metastasis [16, 18, 23] predicting poor prognosis in both entities. These findings strengthen a putative role of ITIH5 as a tumor suppressor in various tumor types, but mechanisms of its function have not been described so far.

In the present study we give clear evidence that the ECM modulator ITIH5 is involved in controlling breast cancer cell migration and colonization in vitro and in vivo. Moreover, ITIH5 drives an epigenetic reprogramming that reverses the aggressive phenotype of basal-like MDA-MB-231 cancer cells to an epithelial-like phenotype involving re-expression of the well-known tumor suppressor gene *DAPK1*.

## Results

### Loss of ITIH5 mRNA expression is predominant in breast tumors of the luminal B, HER2-enriched and basal-type subtype

Previously, we identified aberrant ITIH5 promoter hypermethylation as the molecular cause for its gene inactivation in breast cancer, which was associated with unfavorable prognosis [16]. Therefore, we initially aimed to decipher ITIH5 hypermethylation and its subtype specific expression in a large dataset of The Cancer Genome Atlas (TCGA) [24, 25], in total comprising 1095 different breast cancer samples, 113 normal breast tissues and 7 distant metastases derived from primary breast tumors.

**Fig. 1:**
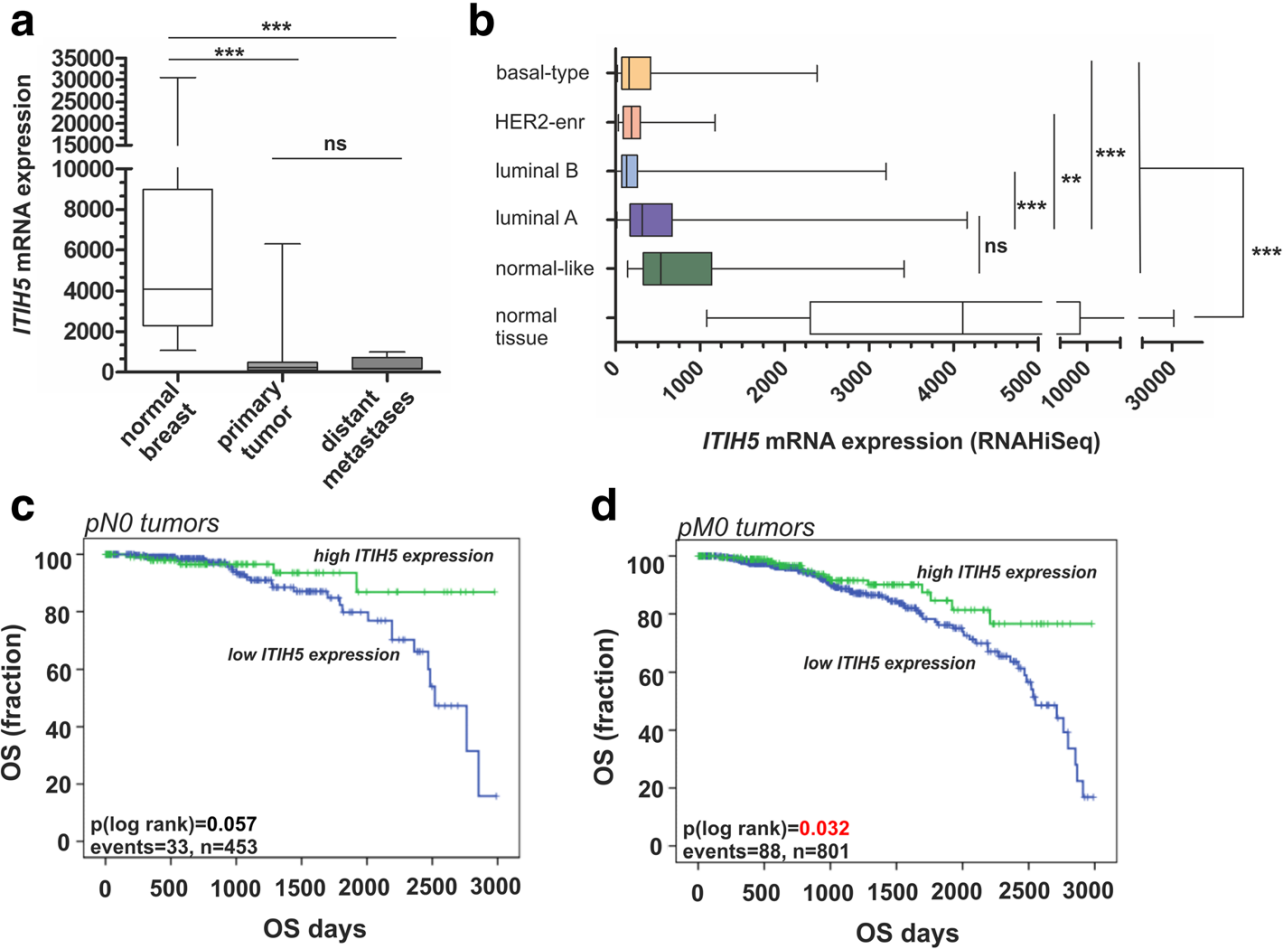
*ITIH5* expression loss in breast cancer subtypes and distant metastases. **a**-**b** Illustration of *ITIH5* mRNA expression based on the TCGA data portal. **a** *Box plot* demonstrating a significant loss of *ITIH5* mRNA expression in primary breast tumors and distant metastases derived from primary breast tumors, *ns*: not significant, ***p < 0.001. **b** Breast tumor samples stratified by subtypes [28], *ns*: not significant, **p < 0.01; ***p < 0.001. **c**-**d** Kaplan-Meier analyses display OS of nodal-negative (pN0, **c**) and non-metastatic (pM0, **d**) breast cancer patients stratified by high *ITIH5* (*green curve*) and low/intermediate *ITIH5* mRNA expression (*blue curve*)

By comparison of breast cancer with healthy control samples, a prevalent loss of ITIH5 expression was found in primary breast tumors (median fold-change (FC): 18-fold downregulation) (Fig. 1a). In distant metastases (n = 7) we still observed a clear absence of *ITIH5* mRNA expression (median FC: 23.5-fold downregulation). Classifying this data set by intrinsic breast cancer subtypes based on Hu et al. [26] we furthermore revealed a pronounced downregulation of ITIH5 mRNA in luminal B (median FC: 31.4-fold downregulation), HER2-enriched (median FC: 22.1-fold downregulation) and basal-like breast cancer (median FC: 25.7-fold downregulation) (Fig. 1b), i.e. breast cancer subtypes known to be associated with high risk for metastasis. In this data set, univariate Kaplan-Meier analyses showed that nodal-negative patients with high ITIH5 expression tend (p = 0.057) to have longer overall survival when compared with low ITIH5 expression (Fig. 1c). In patients lacking distant metastases at initial diagnosis high *ITIH5* expression is significantly (p < 0.05) associated with a longer overall survival when compared with tumors showing low *ITIH5* expression (Fig. 1d).

### ITIH5 promotes apoptosis while suppressing colony growth of breast cancer cells and mediates a morphological shift of metastatic cells in vitro

**Fig. 2:**
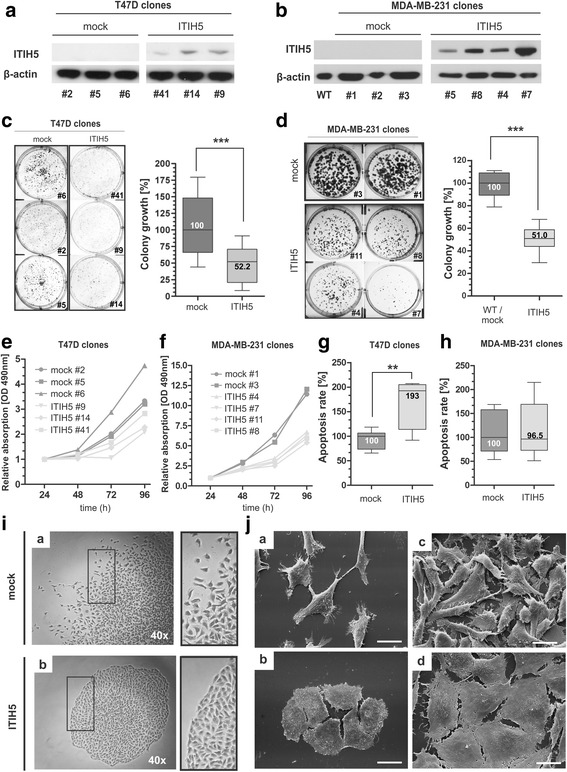
ITIH5 impairs cell growth and colonization of breast cancer cells and induce a phenotype shift in vitro. **a** ITIH5 gain-of-function model of luminal breast cancer cells: Ectopic ITIH5 expression in transfected T47D ΔpBK-ITIH5 clones was confirmed by Western blotting. A specific signal of the ectopic ITIH5 protein is detectable only in T47D ITIH5 clones. β-actin served as loading control. **b** ITIH5 gain-of-function model of basal-type breast cancer cells: Ectopic ITIH5 expression in transfected MDA-MD-231 ΔpBK-ITIH5 single-cell clones was confirmed by Western blotting. A specific signal of the ectopic ITIH5 protein is detectable in MDA-MB-231 ITIH5 clones. β-actin served as loading control. **c** *Colony growth* of luminal T47D breast cancer cells in dependency of ITIH5 re-expression. *Box plot* presents averages of triplicate experiments based on three independent T47D ITIH5 and three T47D mock clones. *Left*: Representative wells with grown ITIH5 as well as mock colonies are shown. *Right*: Densitometrical evaluation of colony growth after 14 days. **d** *Colony growth* of basal-type MDA-MB-231 breast cancer cells due to stable ITIH5 re-expression. *Box plot* presents averages of triplicate experiments based on four independent MDA-MB-231 ITIH5 and two MDA-MB-231 mock clones. *Left*: Representative wells with grown ΔpBK-ITIH5 as well as mock colonies are shown. *Right*: Densitometrical evaluation of colony growth after 14 days. **e**-**f** XTT proliferation assay was performed. T47D **e** and MDA-MB-231 **f** ITIH5 single-cell clones showed reduced cell growth compared with ΔpBK-mock controls. The baseline level at 24 h was set to 1. **g**-**h** Caspase 3/7 activity as indicator of apoptosis in independent T47D **g** and MDA-MB-231 **h** mock and ITIH5 single-cell clones (*n* = 3, respectively). *Box plot* demonstrates relative apoptosis rate. *Horizontal lines*: grouped medians. *Boxes*: 25–75% quartiles. *Vertical lines*: range, minimum and maximum, **p < 0.01. **i** Comparison of morphological MDA-MB-231 colony growth patterns of ITIH5 and mock clones. *Right images*: colony edges. Representative light-micrographs are shown. **j** Comparison of single-cell plasticity showing different confluence of both MDA-MB-231 ITIH5 and mock clones. Representative SEM-micrographs are shown. Scale bar = 20 μm

Addressing the role of ITIH5 in breast cancer, two different in vitro tumor models were generated reflecting both the luminal and basal subtype. Upon stable transfection using a full-length ITIH5 cDNA pBK-CMV expression vector (ΔpBK-ITIH5 clones, also referred to as ITIH5) or the empty vector alone (ΔpBK-mock clones, also referred to as mock), single-cell clones were generated based on well-differentiated, luminal T47D tumor cells (Fig. 2a) and on the metastatic, basal-type MDA-MB-231 breast cancer cell line (Fig. 2b).

At first, the functional impact of forced overexpression of ITIH5 on tumor colony growth was studied using 2D colony formation assays in vitro. Macroscopic analysis of grown colonies clearly visualized reduction in colony size in dependency of ITIH5 overexpression in both (T47D and MDA-MB-231) in vitro models (Fig. 2c and d). Densitometric evaluation of grown colonies significantly confirmed reduced colony growth mediated by ITIH5 expression. Colony formation was suppressed in ITIH5-expressing T47D single cell clones (*n* = 3) by 47.8% (Fig. 2c) and in MDA-MB-231 (*n* = 4) by 49.0% (Fig. 2d) compared to independent mock control clones, respectively. In line XTT proliferation analyses significantly demonstrated reduced cell growth in both cell lines in dependence of ITIH5 overexpression (Fig. 2e and f). Using a caspase-3/7 apoptosis assay, we further showed increased by 92.6% (p < 0.01) programmed cell-death in ITIH5-expressing T47D clones (n = 3 independent clones) compared to mock control cells (n = 3 independent clones) (Fig. 2g). ITIH5 expression had no sustained effect on apoptosis in MDA-MB-231 cells (Fig. 2h). In turn, microscopic analyses revealed fundamental changes in growth patterns of MDA-MB-231 ΔpBK-ITIH5 cancer cells (Fig. 2i and j) but not in T47D transfected cells (data not shown). While mock-transfected MDA-MB-231 cells retained a scattered colony growth, ITIH5-expressing MDA-MB-231 cells formed independently of the amount of forced ITIH5 expression of tested clones (n = 6; Additional file 1) tightly packed colony structures lacking cell spreading at the colony periphery (Fig. 2i). Scanning electron microscopy analyses (Fig. 2j) finally confirmed pronounced morphological changes of independent MDA-MB-231 ITIH5 cell clones at high and low density. ΔpBK-mock cells showed a mesenchymal-like morphology characterized by an elongated cell shape. In contrast, ITIH5-expressing MDA-MB-231 cells grew in a monolayer with a cuboidal single-cell shape indicating a profound impact of ITIH5 action in this metastatic breast cancer cell line.

### ITIH5 suppresses lung colonization by metastatic MDA-MB-231 breast cancer cells in mice

**Fig. 3:**
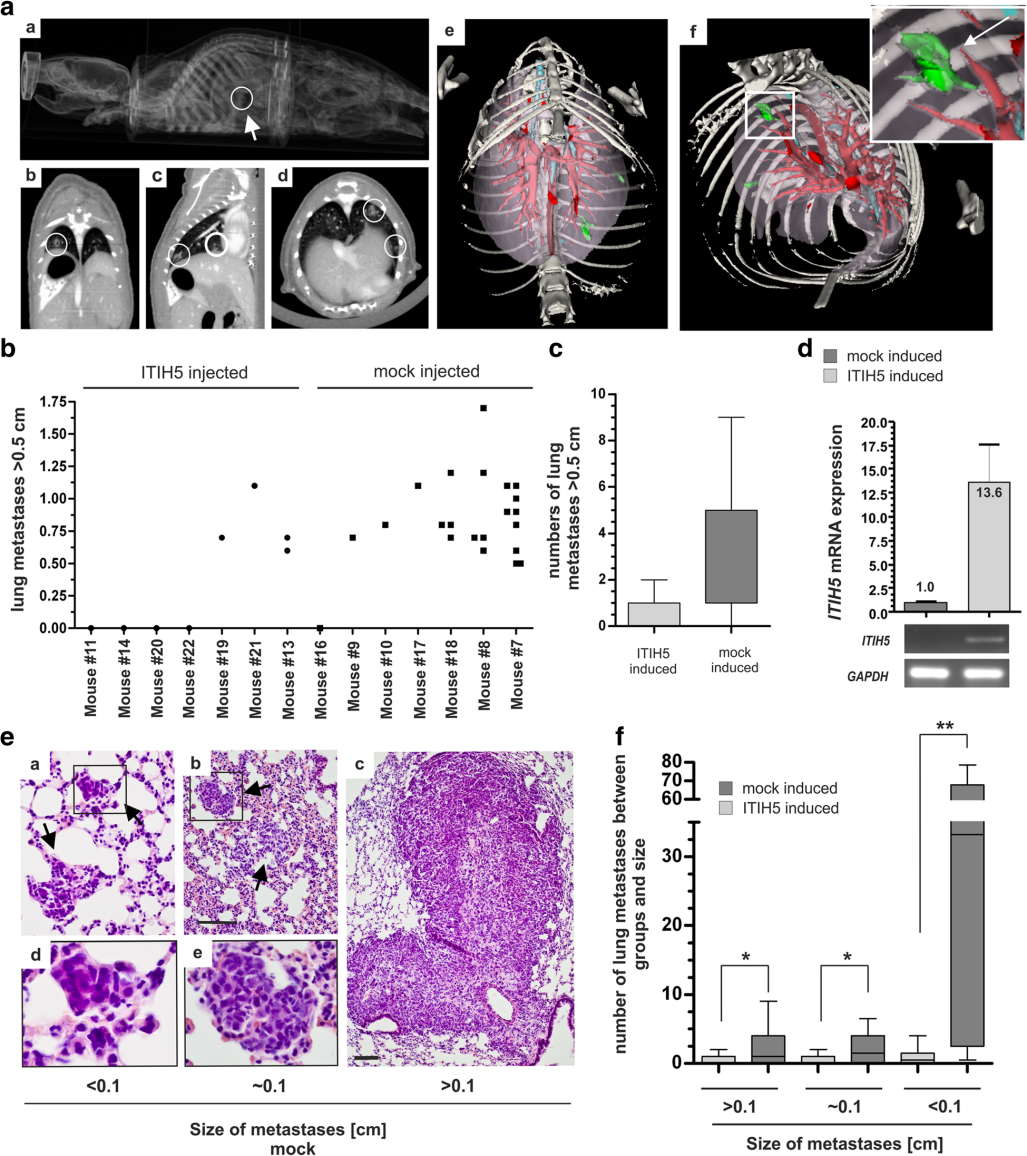
ITIH5 suppresses lung colonization of basal-type breast cancer cells in vivo. **a** In vivo μCT screening highlighted metastatic growth in mouse lungs. Representative 2D (a–d) and 3D (e + f) images of the lung after contrast-agent application and 3D volume rendering are shown. Macro-metastases foci (*white circles*; *green-colored after segmentation*) in mice intravenously injected with MDA-MB-231 mock cells (*control set*) in the pleural space. *Red*: vascular structures. *Blue*: tracheobronchial system. **b** Quantification of metastases by in vivo μCT analyses: Number and nodules size of lung metastases for each mice (*n* = 7) of the ITIH5 set (ITIH5 clones) compared with the control set (*n* = 7) is illustrated. **c** *Box plot* illustrating reduced numbers of grown metastases in mice injected with MDA-MB-231 ITIH5 cells. **d** Human *ITIH5* mRNA in ITIH5-induced lung tumors compared with ΔpBK-mock-induced tumors. *Columns*: Mean of triplicate determinations. *Error bars*, + *standard error of margin* (s.e.m.). **e** Representative H&E stained metastases of each size category of mock-treated animals. *Black arrows*: tumor nodules. Framed rectangle regions are separately enlarged. *Scale bar*: 100 μm. **f** *Box plot* grouped by three metastases size categories verified a decrease of metastasis growth in mice injected with MDA-MB-231-ITIH5 cells (*n* = 7) compared with mice of the control set (*n* = 7), *p* < 0.05, **p < 0.01

To study the putative tumor suppressive function of ITIH5 under physiological conditions, an experimental in vivo metastasis assay was performed using single-cell clones of the highly metastatic MDA-MB-231 cell line. By day 50 after tumor cell injection (i.v.) mice were three-dimensionally (3D) screened using whole-body non-invasive μCT scans to evaluate major organs of metastatic tumor growth (Fig. 3a). No metastases were found in brain or liver, whereas lungs of control mice (injected with MDA-MB-231 ΔpBK-mock single-cell clones) presented high numbers of macro-metastases, i.e. up to 9 metastases/mouse (Fig. 3b and c). Overall 6 out of 7 (85%) mice treated with MDA-MB-231 mock cells showed lung metastases. In comparison to that, the number of macro-nodules in the lung was clearly decreased when mice received MDA-MB-231 tumor cells expressing ITIH5 as confirmed by RT-PCR (Fig. 3d). Only in 3 out of 7 (43%) mice macro-metastases were detected. The highest number of metastases was 2 nodules per mouse (Fig. 3b). Based on histo-pathological assessment of lung sections, we confirmed a clear reduction of macro-metastases by ITIH5 (*p* < 0.05) (Fig. 3e and f). More interestingly, remarkable differences in the number of micro-metastases (<0.1 cm) were found between the ITIH5-group and control mice (Fig. 3f). While control mice exhibited high numbers of micro-metastases (median number: 33.25) that spread over the whole lung tissue, experimental mice receiving ITIH5-expressing tumor cells only presented very low numbers (median number: 0.5) of small tumor nodules.

### ITIH5 remodels ECM composition, enhances cell-matrix adhesion and contractile cell force generation

**Fig. 4:**
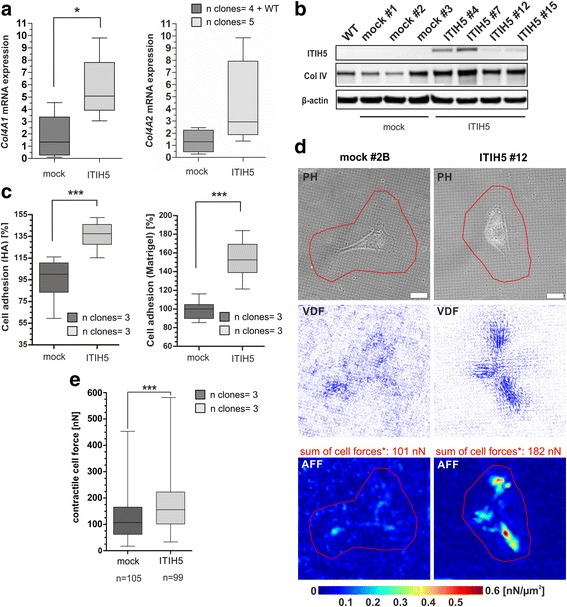
ITIH5 alters ECM-cell interactions and enhances cell-matrix adhesion and contractile cell force generation. **a**-**b** Collagen type IV mRNA and protein (*western blot*) expression in ITIH5 and mock clones. β-actin served as loading control. *p < 0.05. **c** Cell-to-matrix adhesion of ITIH5 and mock clones MDA-MB-231 clones on Matrigel^TM^ and HA-substrate. ***p < 0.001. **d** Cell traction force microscopy assay was used to measure contractile forces that cells exert on elastomeric substrate during cell-matrix adhesion. Representative phase contrast images (PH, *upper row*) of a mock clone and an ITIH5 clone adhered onto elastomeric substrates (15 kPa stiffness) are shown. Vector deformation field images (VDF, *middle row*) were retrieved from fluorescent nanobead displacement tracking Scale bar: 20 μm. Corresponding area force fields (AFF: *lower row*) represent the actual contractile cell force distribution per surface unit (nN/μm^2^). *Red* ROIs: Cell outlines were defined to summarize and compare the whole contractile force exerted by one single cell [nN], *: cell forces of illustrated cells. **e** *Box plots* analysis illustrate the overall comparison of contractile cell force generation of all measured mock clones (1, 2B and #3), and ITIH5-clones (4, 7 and #12). *Box plots*: *Horizontal lines*: grouped medians. *Boxes*: 25–75% quartiles. *Vertical lines*: range, peak and minimum; ***p < 0.0001

So far, data on biological processes and pathways affected by ITI heavy chains besides HA-stabilization are still lacking. Therefore, a transcriptomic micro-array profiling approach was performed followed by gene ontology (GO) annotations using a gene set comparison analysis. Interestingly, over-represented gene annotations confirmed an impact of ITIH5 expression on biological processes (BP) such as “lipid catabolic process” in this metastatic MDA-MB-231 cancer cell line (Table 1) that is in line with recently published data [15]. In addition to those annotations, we further revealed an association of ITIH5 expression with categories like “cell adhesion” or “epithelial cell differentiation”. Interestingly, enrichment of collagens was also shown including upregulation of both transcripts (*COL4A1*, FC: 1.73 and *COL4A2*, FC: 1.53) of the basement membrane (BM) constituent collagen type IV (Table 1). We confirmed increased expression of collagen type IV on mRNA and on protein level in MDA-MB-231 ΔpBK-ITIH5 cells (Fig. 4a and b).

**Table 1:** GO annotated biological processes and cellular components

GO category	GO ontology	GO term	Number of genes	LS permutation *p*-value	KS permutation *p*-value
GO:0016337	BP	cell-cell adhesion	95	**0.00008**	**0.00001**
GO:0016042	BP	lipid catabolic process	56	**0.00022**	**0.00231**
GO:0016339	BP	calcium-dependent cell-cell adhesion	9	**0.00221**	**0.0002**
GO:0030855	BP	epithelial cell differentiation	72	**0.00561**	**0.00337**
GO:0030426	CC	growth cone	23	**0.00037**	**0.00219**
GO:0030427	CC	site of polarized growth	24	**0.00053**	**0.0032**
GO:0005578	CC	proteinaceous extracellular matrix	86	**0.00266**	**0.00134**
GO:0005581	CC	collagen	13	**0.01644**	**0.00332**

Bold *p*-values: significant (*p* ≤ 0.05)

Experimentally, MDA-MB-231 ΔpBK-ITIH5 clones showed altered cell-matrix adhesion dynamics in vitro. On both substrates, i.e. on Matrigel™ that imitates the BM and on HA, ITIH5 expression led to an increased cell-matrix adhesion (Matrigel™: +52.6%, *p* < 0.001; HA: +37.4%; *p* < 0.001) compared to mock control clones (Fig. 4c). Based on this result, cellular traction forces was investigated as potential trigger that could contribute to the modulation of cell behavior, such as enhanced matrix adhesion [27, 28]. For this purpose traction force microscopy (TFM) was used as standard method to quantify contractile forces that cells exert on their surrounding ECM [29, 30]. In order to recapitulate a tumor relevant microenvironment, substrates of 15 kPa stiffness were used for cell adhesion. Such ECM compliance lies in the range of activated breast tumor stroma [31], which results from continuous ECM-stiffening during cancer progression driving invasion and tissue tropism of metastatic tumor cells [32]. In vitro traction force analyses revealed strengthened contractile cell force generation during cell-matrix adhesion of ITIH5 expressing cells (Fig. 4d). The direct comparison with corresponding mock control clones showed a median increase in cell force of 43.9% in MDA-MB-231 ITIH5 clones (ΔpBK-mock: 107.5 nN, ΔpBK-ITIH5: 162.6 nN; *p* < 0.0001) (Fig. 4e).

### ITIH5 modulates integrin signaling that is associated with inhibition of mesenchymal single-cell migration in vitro

**Fig. 5:**
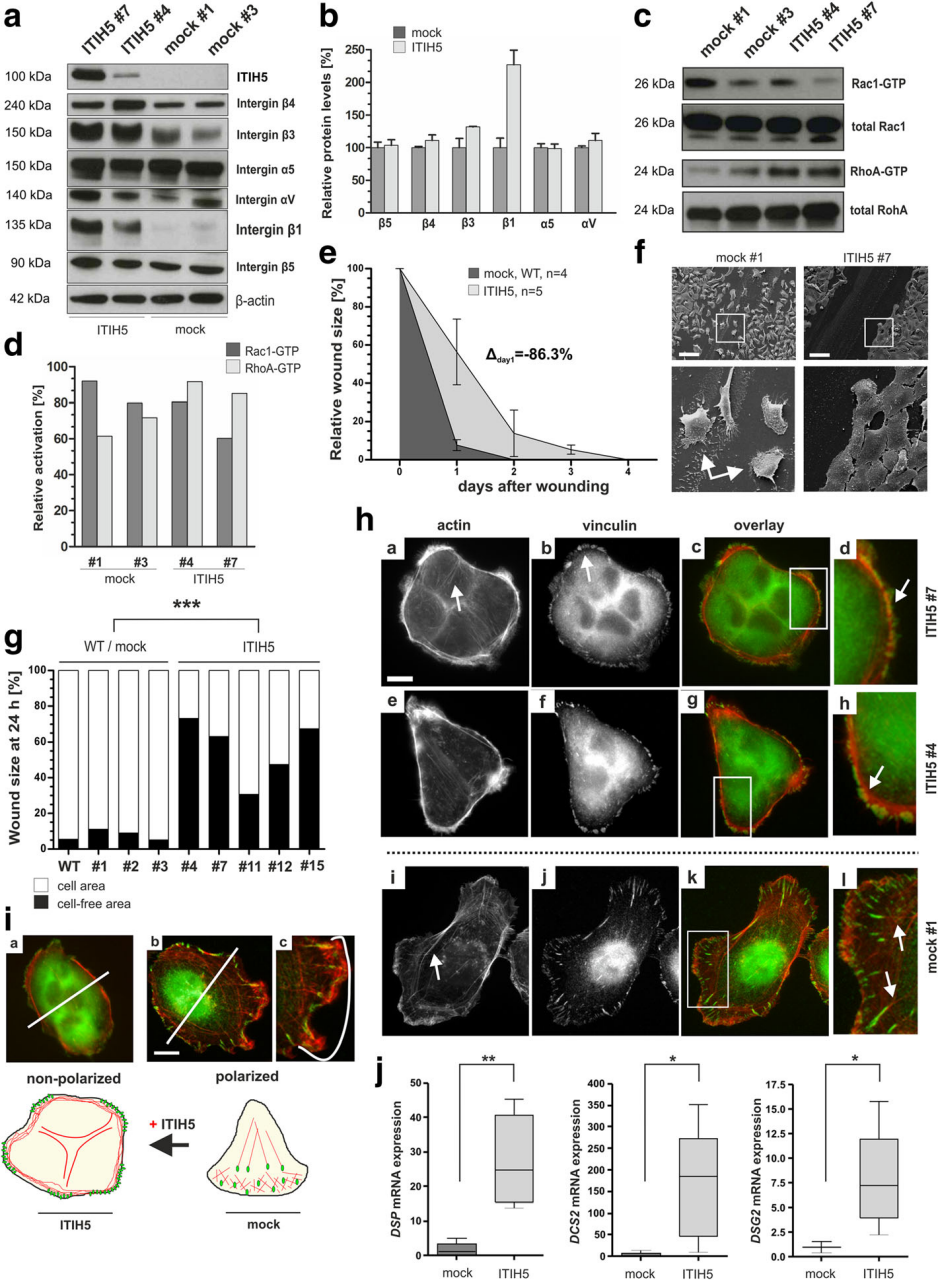
ITIH5 alters integrin signaling impairing single-cell polarization. **a** Integrin protein expression/stability in ITIH5 and mock clones. β -actin served as loading control. **b** Densitometric evaluation of western blot results demonstrating an integrin protein shift. Relative protein expression levels are normalized to β-actin. Mean protein level of mock clones was set to 100%, respectively. **c** Analysis of integrin downstream signaling. Representative western blot results illustrate activated Rac1 and RhoA GTPases in two independent ITIH5 and mock clones. Total Rac1 and RhoA served as loading control. **d** Densitometric evaluation of GTPases activation. Relative protein expression levels are normalized to total Rac1 and total RhoA, respectively. Mean protein level of mock clones was set to 100%. **e** Cell migration was analyzed by using a wound healing assay. Mean migration rate of a control cell set (*n* = 4, WT and mock clones) and ITIH5 MDA-MB-231 clones (*n* = 4) was analyzed over 4 days. *Vertical lines*: *standard deviation* (S.D.) of triplicates. Cell-free area on day 0 was set as 100% and used for standardization. Δ_day1_: differences of cell-free areas on day 1. **f** Documentation of the wounded area by SEM 24 h after scratching. *Left rectangle regions*: separately enlarged. Scale bar = 100 μm. **g** Detailed comparison of wound closure after 24 h for each single-cell clone. **h** Visualization of F-actin architecture and focal adhesion are shown of ITIH5 and mock clones. *Upper rows*: Representative micrographs of ITIH5 clone #7 and ITIH5 clone #4. *White arrows* indicate cortical actin bundles (*red*) and less elongated focal adhesions (*green dots*). *Lower row*: Representative micrographs of mock clone #1. *White arrows*: F-actin stress fibers (*red*) co-localized with elongated focal-adhesion sites (*green*) in the cell body of single-cells. Scale bar = 10 μm. **i** Illustration of the ITIH5-associated impact on cell-polarization necessary for cell migration. *a*: ITIH5 clones showed tight clusters lacking cell polarization. b: mock cells are able to form a distinct protrusive front and a retracting rear. Scale bar = 10 μm **j** Real-time PCR analysis demonstrating significant upregulation of *DSP, DSC2* and *DSG2* in ITIH5 (*n* = 5) compared to mock clones (*n* = 4). *Horizontal lines*: grouped medians. *Boxes*: 25–75% quartiles. *Vertical lines*: range, peak and minimum; *p < 0.05, **p < 0.01, ***p < 0.001

Next, we aimed to decipher the dynamics of the observed mechanical alterations in ECM-cell interactions which just reflects a snapshot of the cell state so far. We focused on integrins that are known to bind ECM, especially, BM components controlling cellular adhesion. Integrins act as anchors by linking the matrix with the intracellular cytoskeleton, while availability of ECM binding sites, i.e. ECM composition and density, has been shown to regulate integrin clustering [33]. In comparison to mock controls, protein level of β3 integrin was increased by 32% and of β1 integrin by 127% in ΔpBK-ITIH5 clones (Fig. 5a and b) thus hypothesizing that ITIH5 expression modulates activation of downstream effectors of the integrin signaling cascade crosstalk like small G-proteins of the Rho subfamily. Activation of the antagonists Rac1 and RhoA was analyzed which has been reported to regulate different steps during cell movement and is thought being modulated by β1 and β3 integrin, respectively [34]. Interestingly, we found that the balance between the activity of RhoA and Rac1 shifted towards increased activation of RhoA in ITIH5 clones while Rac1 was abundantly activated in control cells (Fig. 5c and d).

Consequently a closer look on mesenchymal migration was taken by performing a wound healing assay. Forced ITIH5 expression inhibited cell migration of basal-type MDA-MB-231 cells, i.e. MDA-MB-231 mock clones repopulated the wounded area notably faster than corresponding ITIH5-expressing single-cell clones over 4 days. Impairment of MDA-MB-231 cell migration was confirmed by all analyzed MDA-MB-231 ITIH5 single-cell clones (*n* = 5) compared to the MDA-MB-231 WT and mock clones (*n* = 3). The mean cell motility rate of independent clones of both groups is shown in Fig. 5e. ITIH5-expressing clones were not able to detach from the peripheral edge of the confluent cell layer and to migrate as single-cells into the wound as shown for mock clones (Fig. 5f). Already 1 day after scratching, most mock clones had repopulated almost the entire wound (overall 86.3%), whereas the MDA-MB-231 ITIH5 clones covered on average 43.6% of the wounded area (Fig. 5g). Interestingly, ITIH5 expression did not alter the migration of T47D ΔpBK-ITIH5 single-cell clones (data not shown) whose parental cell line is known to feature already a well-differentiated, epithelial-like phenotype.

Given that, the architecture of the actin cytoskeleton and focal adhesions was determined reflecting integrin clusters on the cell surface of MDA-MB-231 ITIH5 cells. 24 h after seeding ITIH5-expressing MDA-MB-231 cells formed cell clusters whose focal adhesions were found being close to the cell periphery and were less elongated. In contrast mock single-cells exhibited F-actin stress fibers passing through the cell body that are connected with elongated focal adhesion sites in the cell body (Fig. 5h). MDA-MB-231 ITIH5 clones showed less stress fiber formation but formed mostly cortical actin bundles, i.e. the F-actin is condensed around the cell periphery. As expected for those tightly organized cell clusters, single-cell polarization was impaired, i.e. cell polarization into a distinct protrusive front and a retracting rear as obvious in mock cells (illustrated in Fig. 5i). Instead, ΔpBK-ITIH5 cells remained in a tight cell cluster potentially connected by cell-cell contacts as upregulation of desmosomal cadherins was demonstrated. Real-time PCR analysis significantly confirmed increased expression of desmoglein-2 (DSG2, array-effect: FC: 2.04) by 7.2-fold, of desmocollin-2 (DSC2, array-effect: FC: 1.54) by 184.0-fold and of desmoplakin (DSP, array-effect: FC: 1.91) by 24.8-fold (Fig. 5j).

### ITIH5-driven phenotype switch of basal-type breast cancer cells is associated with epigenetic reprogramming

**Fig. 6:**
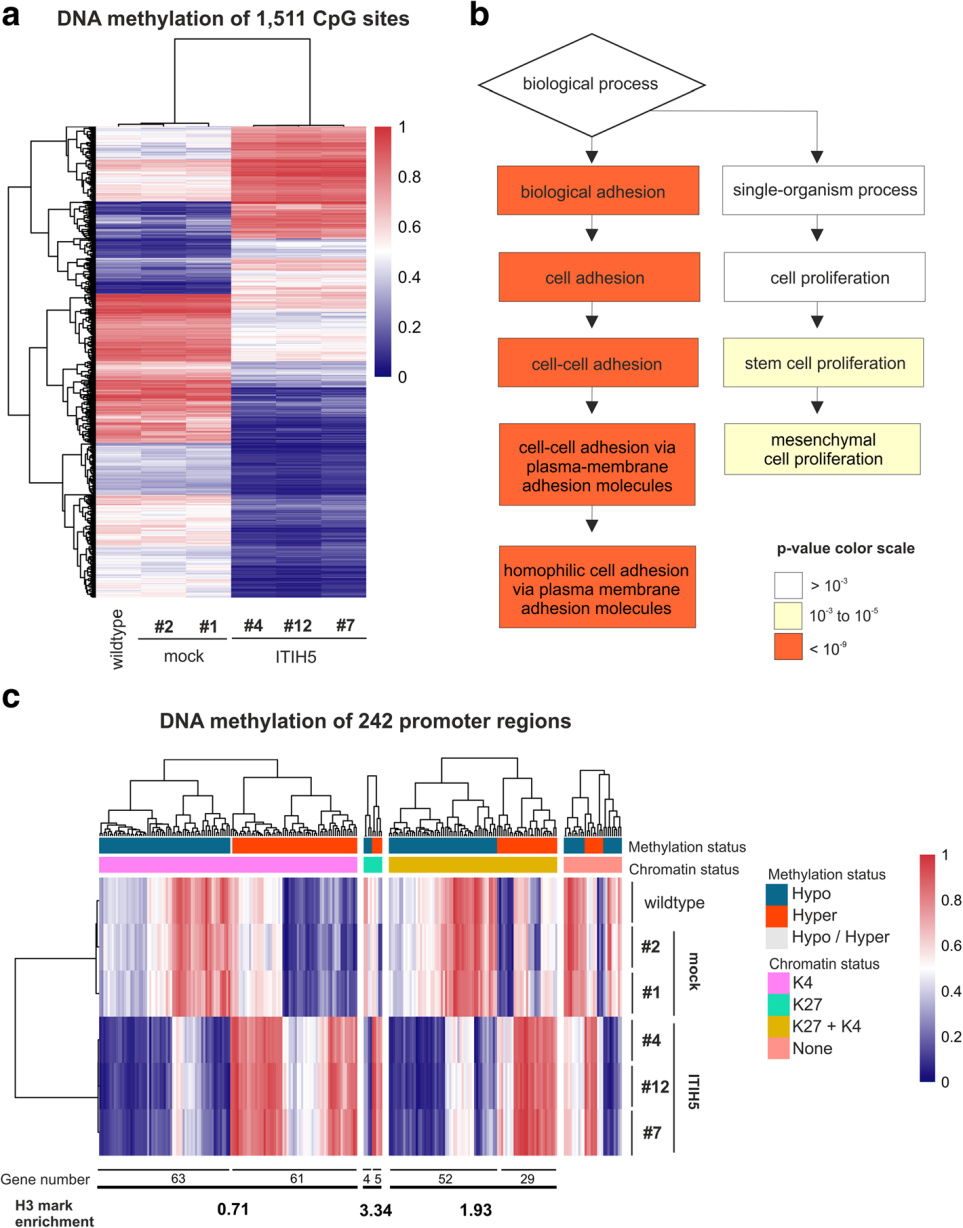
Epigenomic reprogramming of genes involved in cell adhesion and proliferation triggered by ITIH5 expression. Analysis of the DNA methylation profiles of the MDA-MB-231 WT, mock (#1 and #2), and ITIH5 transfected MDA-MB-231 ITIH5 single-cell clones (4, 7, and #12) using the Infinium Human Methylation450 (450 K) BeadChip technology. **a** Heatmap of 1512 CpG sites exhibiting significant (*p* < 0.05, Δβ-value > 0.2) methylation alterations between MDA-MB-231 WT, mock and ITIH5 single-cell clones demonstrated an epigenetic reprogramming of MDA-MB-231 cancer cells. **b** Cartoon illustrating gene ontology analysis of annotated 695 genes corresponding to the 1511 CpG sites. **c** Heatmap analysis of 242 hyper- or hypomethylated CpG sites (out of identified 1511 CpGs) located in potential regulatory promoter regions that are associated with H3K4Me3 and/or H3K27Me3 enrichment in a 5000 bp region upstream and downstream from the TSS. *Chromatin status*: *K27* = H3K27Me3, *K4* = H3K4Me3

Facing the identified phenotype switch of aggressive breast cancer cells driven by ITIH5 expression, we focused on potential mechanisms. It has been suggested that cellular differentiation might impact on epigenetic regulation of gene expression [35]—particularly on DNA methylation [36–38]. Therefore, DNA methylation profiles of the MDA-MB-231 WT, mock (#1 and #2), and ITIH5 single-cell clones (#4, #7, and #12) was analyzed using the Infinium HumanMethylation450 (450 K) BeadChip technology. We then selected those CpG sites with significant (*p* < 0.05) methylation differences (mean β-value) of more than 20% between mock (and WT) and ITIH5-expressing MDA-MB-231 clones: overall 1511 CpG sites passed this threshold (Fig. 6a) corresponding to 728 different genes. 695 of these genes are associated with a GO term including in particular those genes involved in cellular adhesion (e.g. GO:0098742, *p* = 2.98 × 10^−12^) (Fig. 6b). Interestingly, these GO terms were nearly conformable with the GO annotations based on mRNA expression profiling (see Table 1). In addition highly significant enrichment of genes encoding for cellular components of the ECM (e.g. GO: 004442, *p* = 4.68 × 10^−4^) was found including collagens such as *COL2A1* or *COL15A1*. Using gene set enrichment analysis (GSEA) the most significant overlap was observed of hypo- and hypermethylated CpG sites located in a promoter region (TSS1500, TSS200, 5’UTR) (overall *n* = 404) with genes, for instance, 1) containing around the TSS the motif CAGGTG which matches annotation for TCF3 (*p* = 2.26^−20^), or 2) targeted by the Polycomb (PcG) protein SUZ12 (*p* = 1.66^−15^) (Additional file 2).

Subsequently, hyper- and hypomethylated CpGs identified in ITIH5-expressing MDA-MB-231 clones were compared with a list of genes of embryonic stem cells (ES) that have H3K4Me3 and/or H3K27Me3 enrichment in a 5000 bp region upstream and downstream from the transcription start site (TSS) based on previously published ChIP-seq data [39]. The methylation status was determined in 14,356 promoter regions characterized by Ku and colleagues comprising a minimum of 5 CpG sites (Additional file 3). 274 CpG sites out of 1511 were classified according to 242 different corresponding promoter regions (Fig. 6c, Additional file 4). 214 promoters featured a significant (*p* < 10^−6^) association with a potential H3 methylation status described for ES cells. Interestingly, regions associated with the potential PcG signature H3K27Me3 were significantly enriched by 3.3-fold (Table 2) in MDA-MB-231 ITIH5 clones. Promoters with a combined, i.e. with a potentially bivalent, H3K4Me3 and H3K27Me3 status were also enriched by 1.9-fold whereas regions associated with a potential H3K4Me3 status were under-represented in this data set. Hence, the identified epigenetic shift caused by ITIH5 involves promoters potentially associated with bivalent chromatin that may be causative for a dynamic restoration and/or silencing of gene expression.

**Table 2:** Enrichment of differently methylated promoter regions potentially harboring histone H3 modifications described by Ku et al. [39]

	Number of regions	Differential methylated*	Relative enrichment
K4	9118	124 (1.36%)	0.71
K27	140	9 (6.43%)	3.34
K4 + K27	2176	81 (3.72%)	1.93
None	1130	28 (2.48%)	1.29
Total	12,564	242 (1.93%)	

* *p* < 10^−6^ according to Fisher’s exact test

### DNA demethylation of distinct promoter regions is associated with re-expression of the tumor suppressor gene *DAPK1*

We hypothesized that the identified shift in DNA methylation pattern influences expression of genes contributing to the ITIH5 induced, tumor suppressive phenotype of MDA-MB-231 cells. Therefore, we had a closer look on the gene expression pattern associated with ITIH5. By applying a class comparison analysis between control cell populations (mock clones) and ITIH5-transfected clones, we aimed at identifying the strongest co- and anti-regulated genes which met the following criteria: Significantly (*p* < 0.05) differentially expressed with a minimal change in expression by 3-fold. Significantly up- and downregulated genes are summarized in Table 3. While tumor promoting genes such as *AGR2* were downregulated, known tumor suppressor genes like *NDRG2* and *DAPK1* were upregulated 4.3- and 4.6-fold, respectively.

**Table 3:** Genes 3-fold up-/downregulated by ITIH5

Symbol	Fold-change	Parametric *p*-value	Regulation
TNS4	0.13	0.0121	down
CHRDL1	0.21	0.0306	down
AREG	0.22	0.0166	down
AGR2	0.23	0.0169	down
GLB1L2	0.26	0.0455	down
TMEM163	0.29	0.0237	down
ZG16B	0.32	0.0139	down
TPTE	3.45	0.0114	up
NDRG2	4.29	0.0002	up
GJA5	4.34	0.0384	up
GALNT14	4.44	0.0011	up
DAPK1	4.55	0.0485	up
COX7B2	5.05	0.0094	up
PLCB4	5.55	0.0047	up
GMFG	6.51	0.0377	up
THY1	8.31	0.0089	up
ENG	9.00	0.0019	up
LCP1	14.83	0.0121	up

**Fig. 7:**
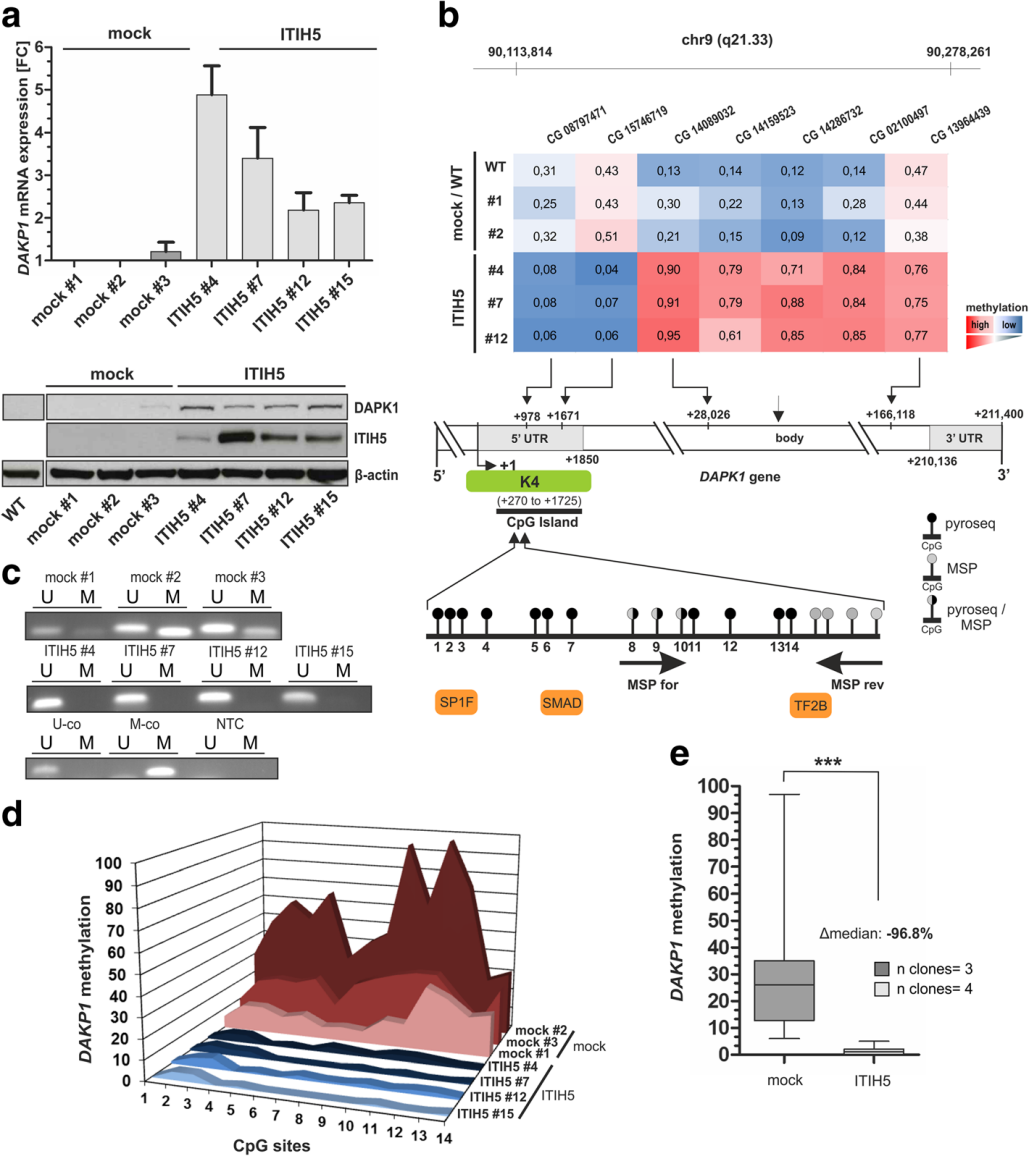
ITIH5 mediates demethylation of the *DAPK1* promoter 5’UTR region leading to its re-expression in basal-type breast cancer cells. **a** DAPK1 re-expression was confirmed in ITIH5 clones (*n* = 4) by real-time PCR *(upper graph)* and western blot analysis *(lower images)* when compared with and mock clones (*n* = 3) and MDA-MB-231 WT. β-actin served as loading control. **b** Schematic map of the human *DAPK1 gene* including the relative positions and β-values of CpG dinucleotides measured by 450 K methylation array profiling in MDA-MB-231 WT cells, mock and ITIH5 single-cell clones. *Red*: high methylation, *blue*: low methylation. +1: *DAPK1* transcription start site (TSS). A predicted CpG island is located between genomic positions 90,112,413 and 90,114,138 (+270 bp to +1725 bp relative to the expected TSS) within the 5’-UTR region. At this site a potential activating H3K4Me3 (K4) histone modification as mapped by Ku et al. [41] was described. The relative positions of 18 CpG sites analyzed either by MSP (used primer: black arrows) and / or pyrosequencing within the *DAPK1* 5’UTR region are indicated. Three putative transcription binding sites in this gene locus were statistically identified: SP1F (matrix similarity: 0.941), SMAD (matrix similarity: 0.963) and TF2B (matrix similarity: 1.0). **c** DNA methylation of the *DAPK1* 5’UTR locus verified in mock and ITIH5 single-cell clones by using MSP. Band labels with U and M represent an unmethylated and methylated DNA region, respectively. Bisulfite-converted unmethylated, (U-co) and polymethylated, genomic (M-co) DNA were used as controls. NTC: non-template control. **d**-**e** Quantification of *DAPK1* 5’UTR DNA methylation frequency by using pyrosequencing. **d** 3D graph illustrates methylation level for each analyzed CpG site (overall 14 CpGs) within the *DAPK1* 5’UTR locus in mock (*n* = 3) and ITIH5 (*n* = 4) single-cell clones. **e** Box plot analysis demonstrates significant reduction of the median methylation ratio within the *DAPK1* 5’UTR region in ΔpBK-ITIH5 compared to ΔpBK-mock clones. *Horizontal lines*: grouped medians. *Boxes*: 25–75% quartiles. *Vertical lines*: range, peak and minimum; *p < 0.05, **p < 0.01, ***p < 0.001

DAPK1 expression was furthermore verified on mRNA as well as on protein level in ΔpBK-ITIH5 breast cancer cells (Fig. 7a). By comparing the profiled DNA methylation of significant CpG sites and the expression signature, a clear demethylation of CpG sites within the 5’ UTR region close to the transcription start site (TSS) of the *DAPK1* gene in ΔpBK-ITIH5 clones (Fig. 7b) was shown. Within this upstream promoter region (ENSEMBL contig ENSG00000196730) a CpG-rich island between genomic positions 90,112,413 and 90,114,138 (+270 bp to +1725 bp relative to the expected TSS) on chromosome 9q was verified which met the following criteria according to Li et al. [40]: DNA region: ≥200 bp; Obs/Exp: ≥0.6; %GC: ≥50. This promoter region corresponds to the 242 identified promoters showing a significantly altered methylation status and is potentially marked by an activating H3K4Me3 histone modification (see Additional file 4). Performing *Genomatix* data base analysis [41] putative transcription binding sites in this 5’UTR locus were determined with highly statistical reliability, namely SP1F (matrix similarity: 0.941), SMAD (matrix similarity: 0.963) and TF2B (matrix similarity: 1.0). In contrast to the 5’UTR region, CpG sites located within the *DAPK1* gene body were clearly hypermethylated when compared to mock control clones (Fig. 7b).

By performing both methylation-specific PCR (MSP) (Fig. 7c) and pyrosequencing (Fig. 7d to e) decreased methylation level within the CpG island closely associated to the TSS of *DAPK1* was subsequently confirmed. Based on pyrosequencing the methylation status of 14 individual CpG sites was analyzed demonstrating completely hypomethylated CpG sites within the 5’UTR region of *DPAK1* in ΔpBK-ITIH5 cells. The median *DAPK1* methylation level of ΔpBK-mock clones (*n* = 3) was 26% featuring a high range between 12.5 and 50.5%, whereas the median methylation of ITIH5 clones (*n* = 4) was consistently decreased in all analyzed ITIH5-expressing clones (*n* = 4) down to 1% (mean: 1.3%, *s.d*. ± 1.4%; range 0.0–1.5%). Overall *DAPK1* methylation was decreased in median by 96.8% (Fig. 7e).

**Fig. 8:**
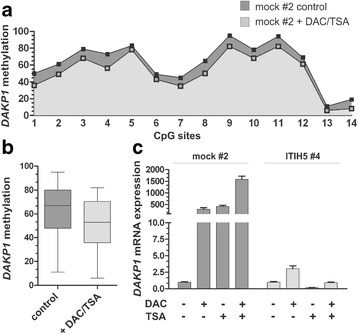
In vitro demethylation of the *DAPK1* 5’UTR locus correlates with *DAPK1* re-expression in ΔpBK-mock cells. **a** Pyrosequencing analysis for each CpG dinucleotide (1–14) within the *DAPK1* 5’UTR region determined prior (−DAC/-TSA; dark-grey-filled) and after in vitro demethylation treatment (+DAC/+TSA; grey-filled). **b** *Box plot* analysis shows reduction of the median methylation ratio within the *DAPK1* 5’UTR region in ΔpBK-mock cells after DAC/TSA treatment (+) compared to non-treated cells (control). *Horizontal lines*: grouped medians. *Boxes*: 25–75% quartiles. *Vertical lines*: range, peak and minimum; **p < 0.01. **c** Real-time PCR results illustrate a clear *DAPK1* re-expression after treatment with both DAC and TSA (+) in mock clones while now further expression of *DAPK1* mRNA was detected in ITIH5 clones already harboring an unmethylated *DAPK1* 5’UTR region. Non-treated cells (-DAC,-TSA) were set to 1, respectively. *Error bars*: + s.e.m

Next, decreased *DAPK1* promoter methylation was demonstrated 72 h after application of demethylation drugs to mock control cells (clone #2). The median methylation level of the analyzed *DAPK1* 5’UTR region was reduced from 67 to 53% (Fig. 8a to b) in mock controls over 3 days. A representative diagram illustrating the methylation level of all 14 analyzed CpGs in mock cells, before and after DAC and TSA treatment is shown in Fig. 8a. As a consequence of the reduced methylation level, upregulation of *DAPK1* mRNA expression was observed in mock tumor cells after demethylation treatment (Fig. 8c) whereas no further *DAPK1* expression was shown in ITIH5 clone #4 harboring already an unmethylated *DAPK1* promoter region as shown in Fig. 7d. In mock control cells, only treatment of both DAC and TSA leads to a maximum of *DAPK1* mRNA re-expression by more than 1500-fold. These findings support our notion that epigenetic alterations of the *DAPK1* promoter may be caused by synergistic crosstalk between DNA methylation and histone modification that has a major impact on the regulation of *DAPK1* re-expression.

### Knockdown of DAPK1 promotes tumor cell migration in MDA-MB-231 ΔpBK-ITIH5 cells

**Fig. 9:**
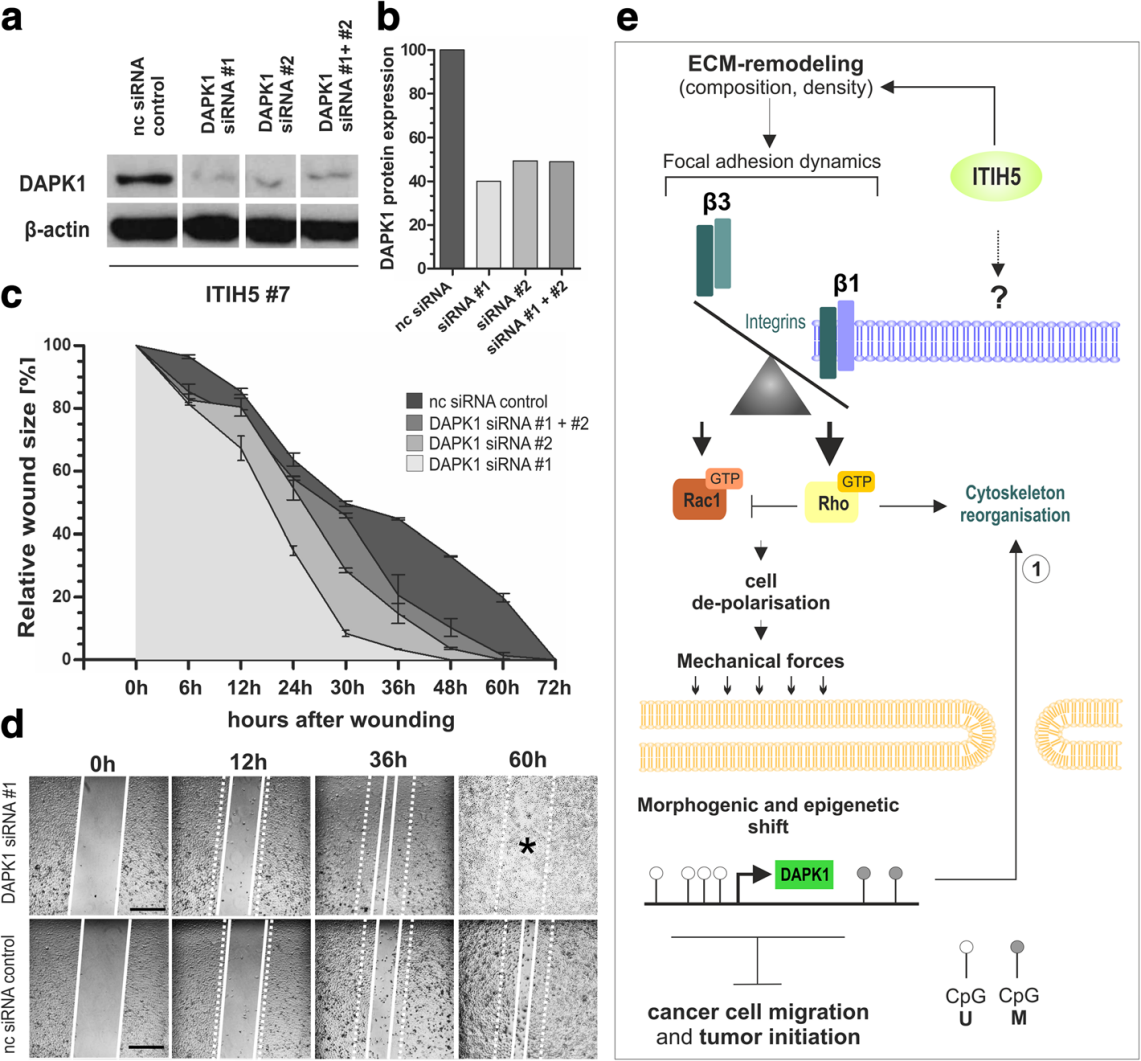
*DAPK1* knockdown restore a motile phenotype in ΔpBK-ITIH5 cells in vitro. **a** DAPK1 protein expression in ITIH5-expressing cells 48 h after transfection with DAPK1-siRNA #1, DAPK1-siRNA #2 as well as #1 and #2 combined in comparison to nc siRNA transfected control cells. β-actin served as loading control. **b** Densitometric determination of DAPK1 protein knockdown in ΔpBK-ITIH5 cells compared to control. **c** Cell migration of ITIH5 clones after treatment with DAPK1-siRNAs was analyzed by using a wound healing assay over 72 h. nc siRNA transfected cells served as negative control. Vertical lines: *standard deviation* (S.D.). Cell-free area on day 0 was set as 100% and used for standardization. **d** Representative wound area documentation by light microscopy of DAPK1-siRNA #1 and nc siRNA control 0, 12, 36, and 60 h after scratching. *White line*: cell-free wound area. *White dashed line*: original wound area size at 0 h. *Scale bar*: 500 μm. **e** Working model highlighting factors potentially involved in ITIH5-driven phenotype shift of mesenchymal MDA-MB-231 breast cancer cells towards an epithelial-like state. ITIH5 remodels the ECM that is accompanied by changes in integrin composition. As a consequence downstream signaling is shifted towards RhoA activation. Clustered cancer cells further lacked polarization but featured in turn strong cell-matrix adhesion and modulated biomechanical cues. Re-expression of DAPK1, caused by epigenetic reprogramming, may be finally involved in ITIH5 mediated suppression of tumor cell migration dynamics potentially by re-organization of cytoskeleton structures as recently described (1): [43, 44]

As DAPK1 (death-associated protein kinase (DAP Kinase)) is a well-known tumor suppressor [42], we aimed to demonstrate whether its re-expression may explain some of the ITIH5-associated suppressive attributes in basal-type breast cancer cells. DAPK1 has been shown to mediate apoptosis but accumulating studies showed involvement of DAPK1 in integrin signaling impairing cell migration [43]. RNA interference-mediated *DAPK1* knockdown was performed in ITIH5-expressing MDA-MB-231 cells (clone #7) applying two different siRNA sequences (#1 and #2) alone as well as in combination (Fig. 9a and b). Interestingly, based on a caspase 3 activity assay, a clear apoptotic resistance of transfected cells was not observed after DAPK1 knockdown (data not shown) that is consistent with a previous report in this cell line [44]. In turn, using a monolayer wound healing assay siRNA-mediated knockdown of *DAPK1* clearly increased tumor cell migration of ΔpBK-ITIH5 clone #7 compared to corresponding cells transfected with the non-silencing control siRNA (nc-control siRNA) that served as negative control (Fig. 9c and d). Stably ITIH5-expressing MDA-MB-231 cells transfected with both *DAPK1* siRNA sequences nearly repopulated the entire wounded cell-free area (siRNA #1: 100%, siRNA#2: 89.71%, siRNA #1+ #2: 96.35%) after 48 h, i.e. ΔpBK-ITIH5 cells with reduced DAPK1 expression tend to restore motile characteristics as observed for MDA-MB-231 WT cells (see Fig. 5g). In contrast to that, nc-control siRNA transfected ITIH5-expressing clones had repopulated only 67.01% of the wound area at this time point. These data clearly demonstrate that the ITIH5-DAPK1 molecular axis plays an important role in the regulation of MDA-MB-231 cell motility.

## Discussion

Previously, we revealed that loss of ITIH5 expression caused by aberrant promoter hypermethylation is associated with poor prognosis and clinical correlates of metastasis in breast cancer [16, 23]. In the current study, ITIH5 downregulation was abundantly found in distant metastases and intrinsic subtypes associated with poor prognosis, i.e. luminal B, HER2-enriched and basal-like breast cancer. ITIH5 loss predicted shorter overall survival of patients with non-metastatic tumors proposing a prominent role of ITIH5 especially in tumors which tend to metastasize early and whose disease management and personalized therapy is still insufficient. To give insight into ITIH5 biology going beyond the assumed role as a prognostic biomarker in breast carcinomas, we established two different stable gain-of-function models, i.e. weak-aggressive T47D and metastatic MDA-MB-231 single-cell clones overexpressing full-length ITIH5. In both cell lines ITIH5 mediated suppression of colony and cell growth while only in luminal-type T47D cells ITIH5-triggered increased programmed cell death. However, this is consistent with our recent finding in luminal-like RT112 bladder cancer cells due to ITIH5 re-expression [18]. These data indicate that ITIH5 may control mechanisms to reduce cancer cell growth independently of a given tumor subtype or entity similar to the described function of ITIH1-3 by stabilizing ECM integrity [9, 45, 46].

In MDA-MB-231 breast cancer cells ITIH5 induced a phenotypic switch, which to our knowledge has not yet been reported for any member of the ITI protein family before. Originally metastatic cancer cells underwent an epigenetic shift driven by ITIH5 that cause a distinct signature of expressed genes. Among others, re-expression of known tumor suppressor genes such as *DAPK1* [42] was clearly demonstrated. As a consequence, forced ITIH5 expression led to a remarkable low-aggressive phenotype causing a reduction of lung colonies in vivo. As metastases were almost exclusively found in lungs of mice injected with cancer cells lacking ITIH5 expression, impaired tumor initiation capabilities could be suggested, a feature mainly attributed to CSC.

Mechanistically, ITIH5 expression was associated with regulation of genes involved in categories of cell adhesion and cell differentiation. Matrix adhesion of ΔpBK-ITIH5 cells was significantly enhanced on physiologically coated substrates, mimicking the basement membrane (BM). ITIH5 also altered the composition of such specialized ECM structures as the BM constituent collagen type IV was identified being upregulated. According to this, profound changes in expression of integrin cell surface receptors were demonstrated that are known to bind to the BM being involved in controlling cell adhesion and migration [34, 47]. Because of their outside-in-signaling capacity, integrins function not only as regulators of cell adhesion but also as sensors of their extracellular environment regulating downstream signaling [48] and it is likely that they have completely different effects on behavior of cancer cells, depending on which integrin receptors and ligands are exposed [49]. Alterations in the profile of integrin expression as identified in ITIH5 clones have been reported to cause dramatic shifts in modes of cell migration [34]. In particular the balance between β1, a putative metastasis suppressor in human cancer [50], and β3 integrin is thought to play a critical role [51]. Interestingly, increased β3 integrin was observed due to ITIH5 re-expression in MDA-MB-231 cells. Nevertheless, β1 integrin, which is almost not expressed in mock clones, is even stronger induced in ITIH5 clones so that the balance between β3 and β1 integrin was clearly shifted towards β1. While β3 integrin has been reported being associated with Rac1 activation, β1 integrin regulates in particular RhoA activity [34]. This notion is important because Rac1 facilitates F-actin polymerization and locally decreases cell-membrane tension that lead to lamellipodia formation during the first step of cell migration. Its activity is blocked by RhoA GTPases in the second phase of cell migration regulating actomyosin contractility [52].

Already in 2005, Danen et al. reported that integrin αVβ3 promotes directional cell migration in the absence of integrin α5β1 being characterized by a single large lamellipodium and lower RhoA activity [53, 54] as also obvious in mock control cells. In turn, α5β1 is particularly efficient at promoting later phases of cell spreading by supporting strong RhoA-mediated contractility and random migration. In our ΔpBK-ITIH5 model we showed that ITIH5-expressing MDA-MB-231 cells were not able to disseminate from neighboring cells moving as single-cells directional into the wounded area. As a consequence ITIH5-expressing significantly higher contractile cell forces compared to their mock clones. This result is in good agreement with the simultaneous upregulation of active RhoA-GTPases in ITIH5 clones, which are known to mediate matrix adhesion-dependent cell forces via Rho/Rock signaling cascades [55] giving a mechanistic explanation for the high-adhesive, well-differentiated phenotype. These findings were associated with clustering of ΔpBK-ITIH5 cells and with reduced polarization into a distinct protrusive front and a retracting rear end. Truong et al. have recently reported that functional inhibition of β1 integrin converted the migratory behavior of human triple-negative breast cancer (TNBC) cells from collective to single-cell movement facilitating lung colonization in vivo [56]. Moreover, β1 integrin promotes an epithelial phenotype in those TNBC cells by restoring, for instance, E-cadherin expression in a TGF-β dependent manner. Hence, upregulation of desmosomal components like DSP and DSC2 linking neighboring cells may contribute to tightly organized colony structures of ITIH5-expressing MDA-MB-231 cells impairing mesenchymal single-cell migration.

It is astonishing that expression of a single ECM factor in vitro, i.e. ITIH5, can effect hyper- or hypomethylation of more than 1500 CpG sites in metastatic cancer cells. The term “epigenetic reprogramming” is commonly used to describe profound alterations in the epigenetic makeup (e.g. [57, 58])—and therefore appears to be justified in this context. Addressing the question why those DNA regions showed differences in DNA methylation, we focused on mechanisms known to be involved in regulating DNA methylation dynamics. So far increasing evidence suggest that histone modifications, namely H3K27Me3 and H3K4Me3, and associated PcG and trithorax-group (trxG) proteins are not only critical for changes in gene expression upon embryonal stem (ES) cell differentiation [59], but also for development of cancer (stem) cells [60–63]. Cross talk between histone methylation marks and DNA methylation is thought to regulate DNA methylation dynamics via recruiting proteins like DNA methyltransferases (DNMTs) [64]. In agreement with that, GSEA analysis revealed highly significant enrichment of genes harboring targets of the Polycomb protein SUZ12. By correlating corresponding CpG positions with histone modification marks as described by Ku et al. [39], 214 promoters were identified that have been previously reported being marked by either H3K4Me3 and/or H3K27Me3 in ES cells and have changed their DNA methylation status in ITIH5 clones. Importantly, genes associated with both H3K27Me3 alone and a combined, i.e. with a potentially bivalent H3K4Me3 and H3K27Me3 status, were significantly overrepresented. Thus, enrichment of promoter regions associated with dynamics in H3 methylation could indeed contribute to the epigenetic shift allowing distinct DNA demethylation patterns as observed for the DAPK1 5’UTR sequence close to the TSS.

DAPK1 is a calmodulin-regulated and cytoskeleton associated serine/threonine kinase [65, 66]. Accumulating evidence suggest that DAPK1 plays an important role in tumor suppression. Epigenetic silencing of *DAPK1* has been demonstrated to correlate with higher risk for recurrence and metastasis in various tumor entities [42]. DAPK1 is a pro-apoptotic factor (e.g. [67]) that abrogates matrix survival signals by inside-out inactivation of β1 integrin impairing the p53-apoptosis pathway [68]. Aside of its apoptotic function Kuo and colleagues postulated an apoptosis-independent mechanism of DAPK1, i.e. uncoupling of stress fibers and focal adhesions by modulation of integrin adhesion [43]. This study fits to our observation that the cytoskeleton was re-organized in DAPK1-expressing ΔpBK-ITIH5 cells. It has been shown that DAPK1 mediates a disruption of the cell polarity by blocking the Rho-GTPases cdc42 in MDA-MB-231 cells leading to inhibition of cell migration in a wound healing assay [44]. Consistent with that, knockdown of DAPK1 had restored motile capacities, at least in part, of ITIH5-expressing MDA-MB-231 cells, indicating involvement of DAPK1 in the RhoA-β1-integrin-mediated signaling axis. A cartoon summarizing these finding is illustrated in Fig. 9e.

Underlying mechanisms of the epigenetic shift induced by ITIH5 in basal-type breast cancer cells and the putative role of specific ECM components and receptors appear complex, and must be addressed in future studies. As luminal T47D cells already grow in epithelial-like clusters, it makes sense that ITIH5 did not trigger a similar effect in those already well-differentiated tumor cells. Beyond that different settings of cell-surface receptors might explain a responsibility for ITIH5-mediated functions such as HA-crosslinking in dependence of a given background. For instance, MDA-MB-231 cells highly express CD44, a known HA-receptor facilitating metastatic CSC-like features [69], whereas T47D has been previously characterized as CD44^low^ [70]. Since Mina Bissell postulated a profound impact of the ECM and regulatory proteins on cell differentiation [1] already in 1982 [71], it is by now well described that epigenetic gene expression control such as chromatin remodeling [2, 72] can be orchestrated by signals from the cellular microenvironment. Biomechanical cues as modified by ITIH5 are thought to contribute to global internal organization of nuclei [73, 74] controlling chromatin structure [36]. Irrespective of that our data underline the complex but fundamental effects of the ECM and its constituents on cell phenotypes and differentiation in the context of malignant progression.

## Conclusions

In the current study, we provide evidence that the ECM modulator ITIH5 suppresses tumor cell migration and colonization of metastatic MDA-MB-231 breast cancer. As a result of an epigenetic reprogramming driven by ITIH5, tumor suppressor genes such as *DAPK1* were re-expressed reversing the aggressive phenotype. Bearing in mind that MDA-MB-231 cells have been shown displaying CSC properties [75, 76], the shift of ITIH5-expressing MDA-MB-231 cancer cells towards an epithelial-like differentiation state accompanied by an inability to initiate high number of metastases in vivo suggests impairment of metastatic characteristics.

## Methods

### Animals

Female BALB/c^nu/nu^ mice were purchased from Charles River Laboratories International (Wilmington, MA). All animal procedures and experiments were conducted in accordance with the German federal law regarding the protection of animals. The respective protocols were approved by the administration of the “Landesamt für Umwelt, Natur und Verbraucherschutz” (LANUV, Recklinghausen, Germany - AZ 87-51.04.2010.A226). For the care of laboratory animals, Guide for the Care and Use of Laboratory Animals (National Institutes of Health publication 86-23, 1985 revision) was followed.

### TCGA data set

Data from breast cancer, normal and metastatic tissues were used from The Cancer Genome Atlas (TCGA) [25], comprising overall patients’ data of an independent platform: Gene expression IlluminaHiSeq (n = 1215). The data of this study can be explored using the cBio Cancer Genomics Portal (http://cbioportal.org).

### Cell lines and reagents

Breast cancer cell lines T47D and MDA-MB-231 were obtained from the American Type Culture Collection (ATCC, Manassas, VA), which assures molecular authentication of cell] lines [77], and was resuscitated before using in experiments. Otherwise cell lines were authenticated, within 12 months of being used in the study and were cultured as described previously [78] and regularly tested for mycoplasma infection using the PCR-based Venor^®^ GeM Mycoplasma Detection Kit (Minerva Biolabs, Berlin, Germany).

### Transfection and single-cell cloning of T47D and MDA-MB-231 cells

Transfection of both T47D and MDA-MB-231 cells with ITIH5-pBK-CMV expression vector, containing the full-length human ITIH5 cDNA derived from normal breast tissue, was performed as recently described [16]. Single-cell clones were selected by limited dilution under geneticin (G418) pressure (T47D: 400 μg/ml; MDA-MB-231: 1000 μg/ml).

### RNA interference of DAPK1

Human ΔpBK-ITIH5 and mock clones were transfected with HiPerfect transfection reagent (Qiagen) applying two siRNA sequences directed against DAPK1 alone (#1: Hs_DAPK1_6, Cat. No. SI02223781, 5’-CGGCTATTACTCTGTGGCCAA -3’ and #2: Hs_DAPK1_6, Cat. No. SI02223774, 5’- AAGCATGTAATGTTAATGTTA.-3’ (20 nM each)), or in combination of both according to the manufacturer’s instructions. Cells were treated every 48 h with siRNA sequences to ensure sufficient DAPK1 knockdown. Commercial non-silencing control siRNA (nc siRNA) (5’-AATGCTGACTCAAAGCTCTG-3’) served as negative control. Knockdown was verified by RT-PCR and western blot analysis after 48, 96 and 144 h. Functional studies were started immediately after 48 h siRNA treatment.

### Nucleic acid extraction and reverse transcription PCR

Total cellular RNA from cultured cells and tumor nodules of mice lungs (samples pooled for test group) was prepared by using TRIzol reagent (Invitrogen). cDNA was synthesized using the reverse transcription system (Promega, Madison, WI) as previously described [16].

### Real-time PCR

cDNAs were amplified by real-time PCR using SYBR-Green PCR mix (Bio-Rad Laboratories, Munich, Germany) performed in an iCycler IQ5 (Bio-Rad Laboratories) and quantified by the comparative C_T_ method calculating relative expression values as previously described [79]. All used primers spanned at least one intron, and are listed in Additional file 5.

### In vitro demethylation

Whole-genome demethylation of human stable MDA-MB-231 clones was performed as recently published [80]. In brief, demethylation agent 5-aza-2’-deoxycytidine (DAC) was added to a final concentration of 5 μM on days 1, 2 and 3. On day 3 cells were additionally treated with 300 nM trichostatin A (TSA) (Sigma-Aldrich). Cells were harvested on day 4 for RNA and DNA extraction.

### Bisulfite-modification and methylation-specific PCR (MSP)

Bisulfite conversion and MSP reaction conditions of in vitro derived DNA was performed as specified previously [81]. For used *DAPK1* MSP primers and cycle conditions see Additional file 6.

### Pyrosequencing

Pyrosequencing of 14 CpG sites within the *DAPK1* 5’UTR region was performed by using the PyroMark PCR Kit (Qiagen) for initial fragment amplification. The PyroMark96 ID device and the PyroGoldSQA reagent Kit (Qiagen) were used as previously described [18]. The DAPK1 assay was designed by using the Pyromark Assay Design Software (Qiagen) and all primers are listed in Additional file 7.

### GTPases pulldown

Activation of both Rac1 and RhoA was measured by using the *Active Rac1 Detection Kit* (#8815, Cell Signaling, Danvers, MA, USA) and the *Active Rho Detection Kit* (#8820, Cell Signaling) respectively, according to the manufacturer’s instructions. In brief, single-cell ΔpBK-ITIH5 and mock clones were cultured in G418 containing growth medium for 48 h. Subsequent to the cell lysis, 550 μg of total cell protein lysate for each clone was mixed with 20 μg of GST-PAK1-PBD capturing (active) RAC1-GTP or GST-Rhotekin-RBD for RhoA. Glutathione matrix-immobilized Rac1-GTP or Rho-GTP was eluted in SDS sample buffer supplemented with DTT. After heat denaturation (5 min, 95 °C) Rac1 and RhoA proteins were detected by western blot analysis using specific antibodies (see Additional file 8). Total cellular RAC1 or RhoA protein was determined for each sample and used for normalization.

### Western blot

Western blot analysis was performed as previously described [82] but slightly modified as following: Proteins were extracted in RIPA lysis buffer, then separated in 4–12% Bis-Tris gels (Invitrogen Life Technologies, Darmstadt, Germany) under reducing (50 mM DTT) conditions using MES-SDS running buffer and electroblotted onto nitrocellulose membranes (0.2 μm). Commercial primary antibodies used are listed in Additional file 8. The generated anti-ITIH5 antibody was previously characterized [18]. Equal protein loading was monitored by using β-actin specific antibody.

### Immunofluorescence

MDA-MB-231-ITIH5 ΔpBK-ITIH5 and mock clones (3 × 10^4^ cells/well) were plated onto 12 mm round glass coverslips. After 24 h incubation, cells were fixed with 4% paraformaldehyde (PFA) and 0.5% Triton X-100 in cytoskeleton buffer (10 mM PIPES, 150 mM NaCl, 5 mM EGTA, 5 mM glucose, and 5 mM MgCl_2_, pH 7.0) for 10 min at room temperature. Afterwards, cells were gently washed twice with PBS and post-fixed with 4% PFA for 10 min at room temperature. Subsequently, cells were washed thrice with cytoskeleton buffer. For vinculin labeling, cells were incubated with the monoclonal antibody hVIN-1 (Sigma-Aldrich, Deisenheim, Germany) for 30 min at room temperature followed by Alexa 488-conjugated goat anti-mouse IgG (Molecular Probes, Eugene, OR). The actin cytoskeleton was labelled with Alexa 594-conjugated phalloidin (Molecular Probes). Coverslips were mounted in Prolong (Molecular Probes). Specimens were observed using an Axiovert 200 microscope (Zeiss, Jena, Germany) equipped with a Plan-Apochromat 100×/1.40 NA oil immersion objective in combination with 1.6× or 2.5× optovar optics. Images were recorded with a cooled, back-illuminated CCD camera (Cascade, Photometrics, Tucson, AZ) driven by IPLab Spectrum software (Scanalytics Inc., Rockville, MD).

### Scanning electron microscopy

Cells were fixed in 3% glutaraldehyde (in 0.1 M Soerensen’s phosphate buffer [13 mM NaH_2_PO_4_ × H_2_O; 87 mM Na_2_HPO_4_ × 2H_2_O; pH 7.4]) for at least 1 h, then rinsed in 0.1 M Soerensen’s phosphate buffer. Next, cells were dehydrated in a graded ethanol series (30, 50, 70, 90, 3% × 100%) and critical-point-dried in carbon dioxide (CPD 010, Balzers Union, FL). The dried samples were fixed on SEM stubs and sputter-coated with gold (SCD 030, Balzers Union), then analyzed with an ESEM XL 30 FEG (FEI Philips, Eindhoven, Netherlands) in high vacuum mode at an accelerating voltage of 10 kV .

### Cell attachment assay

Cell adhesion experiments were carried out as previously described [79] with minor modifications: Six-well plates were coated with HA (100 μg/ml; Sigma-Aldrich) or Matrigel™ (10 μg/ml; Sigma-Aldrich) and cells (5 × 10^5^ cells/well) were incubated to adhere on surface for 30 min at 37 °C. Attached cells were fixed with 70% ethanol for 10 min and stained with 0.1% crystal violet. After 20 min cells were exhaustively washed with water and dried overnight. The dye was dissolved in 0.002% Triton X-100 in 100% isopropanol and carried over into a 96-well plate to measure the optical density at 590 nm using an ELISA reader (SpectraMax 340; Molecular Devices; CA).

### Fabrication of silicone rubber substrates

Substrate preparation and characterization of elastomer material properties (Young’s modulus and Poisson’s ratio) were performed as previously described [83]. In brief, cross-linked elastomeric silicone rubber was used (Sylgard 184, Dow Corning), which is supplied as a two-component kit consisting of base and cross-linker oil. Both components were mixed at a ratio of 1:50 and mixed with 5% (v/v) yellow-green fluorescent nanobeads (0.2 μm diameter, FluoSpheres, Invitrogen). This pre-polymer mixture was applied onto a micro-structured silicon dioxide mold containing 500 nm high microdots with an edge length of 2.5 μm and a lattice constant of 3.5 μm, to generate a regular bead layer within the elastomeric substrate. The polymer layer was then covered by a glass coverslip. A defined layer thickness of 80 μm was produced by putting spacers between the silicon surface and the coverslip. Pre-polymer mixtures were heat cross-linked (60 °C) overnight and finally displayed a Poisson’s ratio of 0.5 and a Young’s modulus of 15 kPa. For cell culture, the silicon mold and spacer were removed and glass bottom covered elastomer substrates were glued to a 3.5 cm Petri dishes with 1.5 cm holes.

### Traction force microscopy and cell force retrieval

Live cell analyses were performed at 37 °C and 5% CO_2_ (cell incubator XL2, Carl Zeiss, Germany) using an inverted confocal laser scanning microscope (cLSM710, Carl Zeiss, Germany), utilizing a 40× EC Plan-Neofluar oil immersion objective (PH3, NA = 1.3, Carl Zeiss, Germany). Images were taken using the imaging software ZEN 2.1, Carl Zeiss Germany). Confocal micrographs of the cells (phase contrast) and of yellow-green fluorescent beads were taken using an argon ion laser (488 nm) with a transmitted light detector and a 490–530 nm bandpass filter, respectively. Cells were seeded onto fibronectin-coated (20 μg/cm^2^) TFM substrates 48 h before measurement. Only well-adhered cells were analyzed. Traction forces applied by a single cell to an elastic substrate of defined stiffness cause deformations fields that were visualized by tracking fluorescent marker beads in the substrate. From the displacement of these particles cell forces were calculated. Substrate deformation was captured in the presence of cells and substrate relaxation was obtained after cell elimination by trypsinization. Cell area force fields (AFF) were retrieved from vector displacement fields (DVF) determined by correlating the nanobead displacement in the deformed and the relaxed, cell-free elastomer. MatLab-based algorithms were used for data processing as previously described [29, 84].

### XTT cell proliferation assay

The XTT proliferation assay (Roche Diagnostics, Mannheim, Germany) was used and performed as previously described [16].

### Apoptosis assay

Activity of the effector caspases 3 and 7 in ITIH5 and mock single-cell clones was analyzed by using the Apo-One® Homogeneous Caspase-3/7 Assay (Promega, Mannheim, Germany) according to the manufacturer’s instructions. Briefly, cells (1.5 × 10^4^) were seeded in 96-cell culture wells and incubated overnight (20% O_2_, 5% CO_2_, 37 °C). Afterwards, staurosporine (1 μM, Sigma-Aldrich, Deisenhofen, Germany) was applied to induce apoptosis. Fluorescence intensity was quantified by using an ELISA plate reader (excitation: λ = 485 nm; emission: λ = 577 nm).

### In vitro colony formation and migration studies

Colony formation assays were performed as previously described [79]. In vitro motility was analyzed performing a monolayer scratch wound assay as previously specified [85].

### In vivo metastasis assay

MDA-MB-231 cells (3 × 10^6^) of the ITIH5 test set (ΔpBK-ITIH5 clones) or the control set (ΔpBK-mock clones) were intravenously inoculated into the lateral tail vein of 7 week old female Balb/c^nu/nu^ mice. After 50 days, mice were μCT scanned, and then sacrificed. Lungs were harvested, photographed with the Discovery V12 stereomicroscope (Zeiss), analyzed with DISKUS software package (Königswinter, Germany), formalin-fixed (10%) and paraffin-embedded. H&E-stained sections from each lung tissue as well as a further slide sectioned at 30 μm increments in the vertical plane were examined by a pathologist in a blinded manner to quantify the number of micro-metastases.

### In vivo micro-computed tomography

Whole-body scans of mice were performed using non-invasive μCT. A gantry-based dual energy micro-computed TomoScope 30s Duo (CT Imaging, Erlangen, Germany) was used. Matched pairs of mice (*n* = 7 each) were scanned 50 days after tumor cell injection and anaesthetized using a 1.5% isoflurane inhalation narcosis. Mice were scanned both natively and after intravenous application of eXIA™160 (Binitio Biomedical, Ottawa, Canada), an iodine-based and radiopaque blood pool contrast agent. Injected dose of 0.1 ml/20 g body weight was used [86]. Images were reconstructed using a Feldkamp type reconstruction (CT-Imaging, Erlangen, Germany) generating a voxel size of 70 × 70 × 70 μm^3^. Subsequently, images were analyzed using Amide [87]. 3D architecture was visualized using Imalytics Preclinical software [88].

### Gene expression profiling

Gene expression profiling of the ITIH5 test set (three independent MDA-MB-231 ΔpBK-ITIH5 clones) and the control set (three independent MDA-MB-231 ΔpBK-mock clones) was carried out by the IZKF Chip-Facility (Interdisciplinary Centre for Clinical Research Aachen within the Medical faculty of the RWTH Aachen University) using the Affymetrix 1.0 ST gene array (Affymetrix, Santa Clara, CA).

Profiling of stably transfected MDA-MB-231 breast cancer cells was performed using BRB-ArrayTools developed by Dr. Richard Simon and BRB-ArrayTools Development Team version 4.3.0 – Beta. In order to identify the significantly regulated candidate genes the *class comparison* evaluation was used [89], which met the following criteria: Significantly (*p* < 0.05) differentially expressed with a minimal change in expression by 3-fold. Exact permutation *p*-values for significant genes were computed based on 35 available permutations. Genes were excluded when less than 20% of expression data had at least a 1.5-fold change in either direction from gene’s median value. Gene Ontology (GO) categories were determined by applying a gene set comparison analysis that is similar to the gene set enrichment analysis described by Subramanian et al. [90]. Tests used to find significant gene sets were: LS/KS permutation test (to find gene sets which have more genes differentially expressed among the phenotype classes than expected by chance). Over-represented GO lists were considered significant when the threshold of determining significant gene sets is equal or below 0.005 (LS/KS permutation test).

### DNA methylation profiling

DNA methylation profiles were analyzed in three independent MDA-MB-321 ΔpBK-ITIH5, two mock clones and WT by using the HumanMethylation450 Beadchip technology (Illumina, San Diego, USA). Hybridization of bisulfite converted DNA (200 ng) and initial data evaluation was performed by the DKFZ Gene Core Facility (Heidelberg, Germany).

Limma-*T*-test statistics was calculated in *R* [91] to select for CpG sites with significant differences in DNA methylation (adjusted *p* value <0.05 and 20% differential DNA methylation level between both test groups). Cluster analysis of the CpG sites was performed with the “pheatmap package” for R using complete linkage and Euclidean distance [92]. The Gene Ontology analysis was performed using the *GOrilla* software tool to visualize GO terms of target (1511 GpG sites) and background list (all analyzed CpG sites) [93]. Overlap of significantly hyper- and hypomethylated CpG sites between ΔpBK-ITIH5 and ΔpBK-mock clones with gene set data bases was performed using a public gene set enrichment analysis platform (GSEA; http://www.broadinstitute.org/gsea/index.jsp) [90, 94]. The probes / CpG sites of the HumanMethylation450 BeadChip were furthermore annotated with previously published data on the presence of two histone H3 modifications (H3K4Me3 and H3K27Me3) close to a transcription start site in embryonic stem cells [39]. We used the information on the probed location (GRC36 reference) provided by the manufacturer (HumanMethylation450 v1.2 Manifest File). A promoter region that contained at least one probed CpG site with a significant difference in DNA methylation level was called deregulated (Additional file 3). The subsequent analysis was limited to the 12,564 (69%) regions with a minimum of 5 probed CpG sites to reduce the bias introduced by a low coverage. Methylation β-values of multiple significant different methylated CpG sites were averaged after transformation to M-values.

### Statistics

Statistical analyses were performed using GraphPad Prism 5.0 (GraphPad Software Inc., La Jolla, CA) and SPSS 20.0 (SPSS, Chicago, IL). Differences were considered statistically significant if the two sided *p*-values were equal or below 5% (≤0.05). To compare two or more groups the Mann-Whitney or Kruskal-Wallis test was used, respectively. Overall survival (OS) was measured from surgery until death and was censored for patients alive at the last follow-up using the univariate log-rank tests.

## Additional files

Additional file 1: Cell plasticity of ITIH5-expressing MDA-MB-231 single-cell clones. This figure shows morphological characteristics of independent MDA-MB-231 single-cell clones using phase-contrast microscopy. (DOCX 109 kb)
Additional file 2: Overlap of hypo-and hypermethylated CpG sites with published gene sets (GSEA). This table summarizes an overlap of significantly hyper- and hypomethylated CpG sites between ΔpBK-ITIH5 and ΔpBK-mock clones with gene set data bases. (XLS 72 kb)
Additional file 3: CpG sites with potential H3 marks. This table lists CpG sites of the HumanMethylation450 BeadChip annotated with histone H3 modifications (H3K4Me3 and H3K27Me3). (XLSX 7866 kb)
Additional file 4: Identified promoter regions with potential H3 marks that are hyper- or hypomethylated due to ITIH5 expression. This table illustrates the 242 different promoter regions featuring a significant association with a potential H3 methylation status described for ES cells. (XLSX 23 kb)
Additional file 5: Primer sequences and reaction conditions for RT-PCR experiments. This table illustrates the sequences of all primers used in this study for RT-PCR analysis. (XLS 28 kb)
Additional file 6: Primer sequences and PCR conditions for MSP analyses. This table illustrates the sequences of all primers used in this study for MSP analysis. (XLSX 10 kb)
Additional file 7: Primer sequences for pyrosequencing. This table shows sequences of DAPK1 pyrosequencing. (XLSX 10 kb)
Additional file 8: Primary antibodies used in this study. This table lists all primary andtiboides used in this study. (XLS 26 kb)

## Abbreviations

2D: Two-dimensional
3D: Three-dimensional
AFF: Area force field
ATCC: American Type Culture Collection
BM: Basement membrane
BP: Biological process
CA: California
cDNA: Copy number desoxyribonucleic acid
CMV: Cytomegalovirus
COL4: Collagen 4
CpG: 5’-Deoxycytidine-phosphate-deoxyguanosine-3’
CSC: Cancer stem cell
DAPK1: Death-associated protein kinase 1
DNA: Deoxyribonucleic acid
DNMT: DNA methyltransferase
dNTP: Desoxyribonucleosidtriphosphate
DSC2: Desmocollin 2
DSG2: Desmoglein 2
DSP: Desmoplakin
ECM: Extracellular matrix
EK: Ethics committee
EMT: Epithelial-to-mesenchymal transition
ES: Embryonic stem cell
FA: Focal adhesion
FC: Fold change
FCS: Fetal calf serum
FFPE: Formalin fixed paraffin embedded
GAPDH: Glyeradehyde 3-phosphate dehydrogenase
GO: Gene ontology
GSEA: Gene set enrichment analysis
GTP: Hydrolyze guanosine triphosphate
h: Hour
H3K27me3: Trimethylation mark at K27
H3K4me3: Trimethylation mark at K4
HA: Hyaluronan
HABP: HA-binding protein
i.v.: Intravenous
IL: Illinois
ITI: Inter-a-trypsin inhibitor
ITIH: Inter-a-trypsin inhibitory heavy chain
IZKF: Interdisziplinäres Zentrum für Klinische Forschung
LANUV: Landesamt für Umwelt, Natur und Verbraucherschutz
MEF: Embryonic fibroblast
min: Minute
mRNA: Messenger ribo nucleic acid
MSP: Methylation specific PCR
n: Number
nN: Nano Newton
NPC: Neural precursor cell
PBS: Phosphate-buffered saline
PcG: Polycomp-group
PCR: Polymerase chain reaction
ROI: Region of interest
RT: Room temperature
RT-PCR: Real-time PCR
RWTH: Rheinisch-Westfälisch Technische Hochschule
s.e.m.: Standard error of the margin
siRNA: Small interfering RNA
TCGA: The Cancer Genome Atlas
TFM: Traction force microscopy
TGF: Transforming growth factor
trxG: Trithorax-group
TSS: Transcription start site
USA: United States of America
UTR: Untranslated region
VA: Virginia
VDF: Vector deformation field
VIT: Vault protein inter-α-trypsin
vWA: Von Willebrand A
WI: Wisconsin
WT: Wildtype
μCT: Micro-computed tomography
