# Supplementary material for: ITIH5 mediates epigenetic reprogramming of breast cancer cells
Source: Mol Cancer. 2017 Feb 23;16:44. doi: 10.1186/s12943-017-0610-2 (PMC5322623; doi:10.1186/s12943-017-0610-2)
Supplement: Additional file 1: — Cell plasticity of ITIH5-expressing MDA-MB-231 single-cell clones. This figure shows morphological characteristics of independent MDA-MB-231 single-cell clones using phase-contrast microscopy. (DOCX 109 kb) [file 12943_2017_610_MOESM1_ESM.docx]

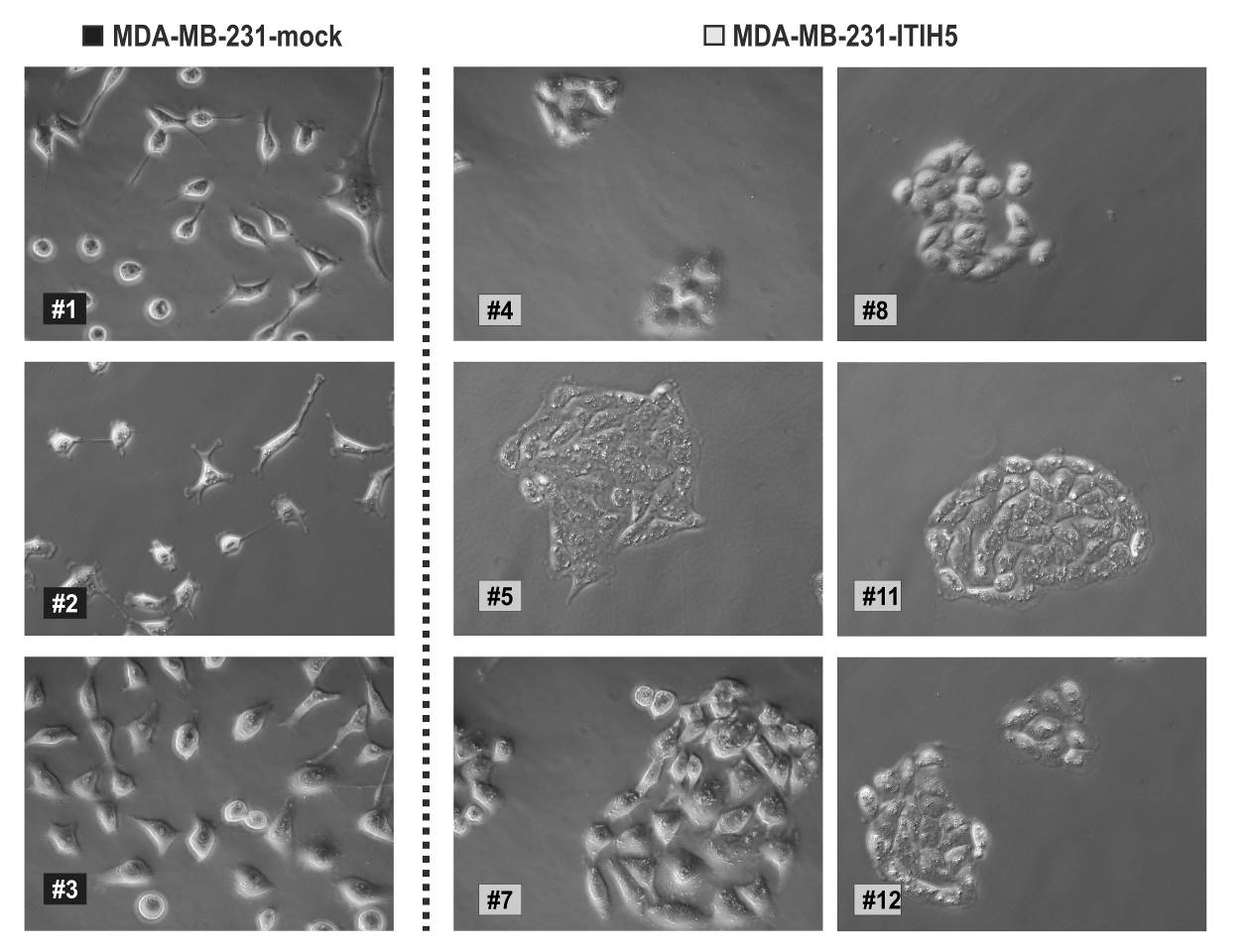


**Additional file 1:** Morphological characteristics of independent MDA-MB-231 single-cell clones (ΔpBK-mock n=3; and ΔpBK-ITIH5 n=6) using phase-contrast microscopy (original magnification x200).
